# Finding disease modules for cancer and COVID-19 in gene co-expression networks with the Core&Peel method

**DOI:** 10.1038/s41598-020-74705-6

**Published:** 2020-10-19

**Authors:** Marta Lucchetta, Marco Pellegrini

**Affiliations:** 1 Institute of Informatics and Telematics (IIT), CNR, Pisa, 56124 Italy; 2 Department of Biotechnology, Chemistry and Pharmacy, University of Siena, Siena, 53100 Italy

**Keywords:** Computational biology and bioinformatics, Diseases

## Abstract

Genes are organized in functional modules (or pathways), thus their action and their dysregulation in diseases may be better understood by the identification of the modules most affected by the disease (aka disease modules, or active subnetworks). We describe how an algorithm based on the Core&Peel method is used to detect disease modules in co-expression networks of genes. We first validate Core&Peel for the general task of functional module detection by comparison with 42 methods participating in the Disease Module Identification DREAM challenge. Next, we use four specific disease test cases (colorectal cancer, prostate cancer, asthma, and rheumatoid arthritis), four state-of-the-art algorithms (ModuleDiscoverer, Degas, KeyPathwayMiner, and ClustEx), and several pathway databases to validate the proposed algorithm. Core&Peel is the only method able to find significant associations of the predicted disease module with known validated relevant pathways for all four diseases. Moreover, for the two cancer datasets, Core&Peel detects further eight relevant pathways not discovered by the other methods used in the comparative analysis. Finally, we apply Core&Peel and other methods to explore the transcriptional response of human cells to SARS-CoV-2 infection, finding supporting evidence for drug repositioning efforts at a pre-clinical level.

## Introduction

In a typical systems biology paradigm, large amount of molecular data collected via high throughput ’omics’ experiments are stored in curated databases then filtered and reorganized in the form of an interaction network among molecular species (for example, co-expression networks are built via measures of the co-expression of genes under a variety of conditions)^1,2^. Next, such a network is analyzed to detect interesting phenomena from a biological point of view, potentially relevant for a phenotype of interest or a specific biological process. Biological networks have been found to have a rich modular structure which mediates biological processes and cellular activities. Thus discovering and validating modules within biological networks has become an activity propaedeutic to the discovery of biological mechanisms. Genes in a functional module should act in a highly correlated (or anti-correlated) way in response to cell conditions, as revealed by approaches integrating different levels of ’omic’ data^3^.

In a second paradigm typical of systems biology , we are not so much interested in finding genes that behave similarly across a variety of cell conditions, but in finding genes (or modules) whose behavior is different between two clearly stated cell conditions to find subnetworks that are activated differently and thus characterize at a functional level the differences between the two conditions (*active subnetwork detection problem*) (^4–8^).

Module and active module identification in biological networks is a key component of a full network analysis aimed at exploring issues related to applications such as drug target discovery^9,10^, cancer subtypes classification^11^, finding biomarkers for cancer prognosis^12,13^, detection of histone modifications^14^, and many others. Previous work on active subnetwork analysis in cancer has focused on finding similarities among cancer subtypes and detecting of prognostic discriminative subnetworks. Gaire et al.^15^ apply subnetwork analysis to Breast Cancer (BC) data looking for conserved circuits in BC subtypes; their finding suggest that subtypes of different cancers may have molecular similarities indicating that therapeutic approaches in different sub-types of cancer may be shared. Chuang et al.^16^ propose a subnetwork-based analysis of gene expression profiles to discriminate between groups of patients with various risks for chronic lymphocytic leukemia (CLL) progression. For COVID-19, active subnetwork analysis has been mainly focused on helping current drug repositioning efforts^17–19^. For a general literature digest on COVID-19, see Harapan et al.^20^.

**Figure 1:**
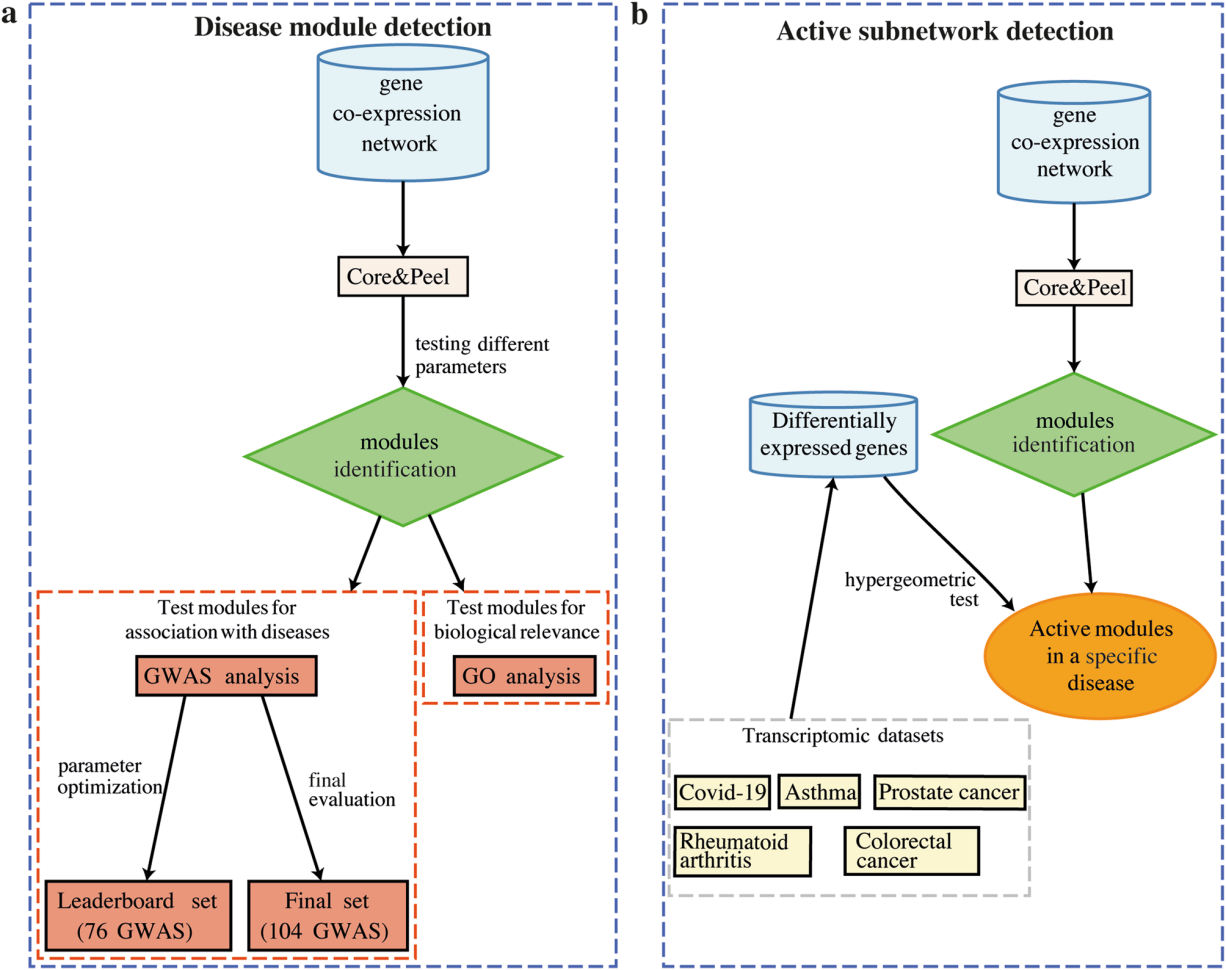
Schematic workflow illustrating the main steps used in our work. Core&Peel was tested for two problems: (**a**) disease module identification and (**b**) active subnetwork detection. In both tasks, the DREAM challenge gene co-expression network was used as input of Core&Peel algorithm. In (**a**) we tested different values of density and Jaccard index. For each parameters combination, we tested the disease-association of each module detected by Core&Peel using the GWAS datasets made available by the DREAM challenge. The leaderboard set (including 76 GWAS datasets) was used to optimize the parameters and the final test (including 104 GWAS datasets) to get a final evaluation of them. We also tested the biological relevance of each module through the Gene Ontology (GO) enrichment analysis. In (**b**), we used the differentially expressed genes and the hypergeometric test to select the active Core&Peel modules in a specific disease. We used several transcriptomic datasets: two cancer RNA-Seq studies (prostate and colorectal cancer), two inflammatory disease microarray experiments (asthma and rheumatoid arthritis) and three COVID-19 related datasets. We used the R/CRAN package *DiagrammeR*^96^ to illustrate the workflow.

This paper demonstrates the performance of the Core&Peel method for these two key problems in systems biology: (a) network module identification and (b) active subnetworks detection. We illustrate the complete workflows in Fig. 1a,b for the two problems, respectively. In both cases, we used the same gene co-expression network as Core&Peel input. In the first task (Fig. 1a), the output modules have been tested for association with diseases using two sets of genome-wide association studies (GWASs) as well as Gene Ontology (GO) enrichment analysis for assessing a biological relevance. In the second task (Fig. 1b), we selected only the Core&Peel modules significantly enriched for the differentially expressed genes to obtain active subnetworks in two cancer types (prostate and colorectal cancer), two inflammatory diseases (asthma and rheumatoid arthritis) and COVID-19 infection.

We show that Core&Peel has a performance that matches or surpasses that of current state-of-the-art approaches, in several measurements, on benchmark diseases, in particular cancer benchmark datasets. Finally, we apply Core&Peel and other state-of-the-art methods to explore the transcriptional signature of the response of human cells to SARS-CoV-2 infection at a modular level. The combined output of these algorithms uncovers several enriched pathways related to current drug repositioning efforts.

The Core&Peel method has been developed in^21^ for predicting protein complexes in large protein-protein interaction networks (PPIN). Core&Peel follows a generalist approach, assuming that modules have the topological properties of quasi-cliques and ego-networks. The method makes a very mild assumption on the properties of the input network, and of the predicted modules, thus in principle, it can be applied quite directly to other community detection problems with a similar flavor. One example of such a problem is disease module identification. In 2016, an open DREAM challenge attempted to identify the best performing algorithms for disease module identification^22^. We applied our previously reported Core&Peel algorithm^21^ to the DREAM challenge data to see how well Core&Peel could perform on this new task. In practice, the list of 42 methods mentioned in^22^ for functional module detection, and the list of 19 methods mentioned in^21^ for protein complex prediction, have very little overlap; the only methods mentioned in both lists are SPICi and ClusterOne, which are used in the functional module detection DREAM challenge as part of an ensemble or hybrid computational pipelines. Thus the nature of the two problems, functional module identification and protein complex prediction, is such as to require a fresh look as it is not obvious that any algorithm can perform equally well in either case.

## Results

### Core&Peel: detection of disease modules

**Figure 2:**
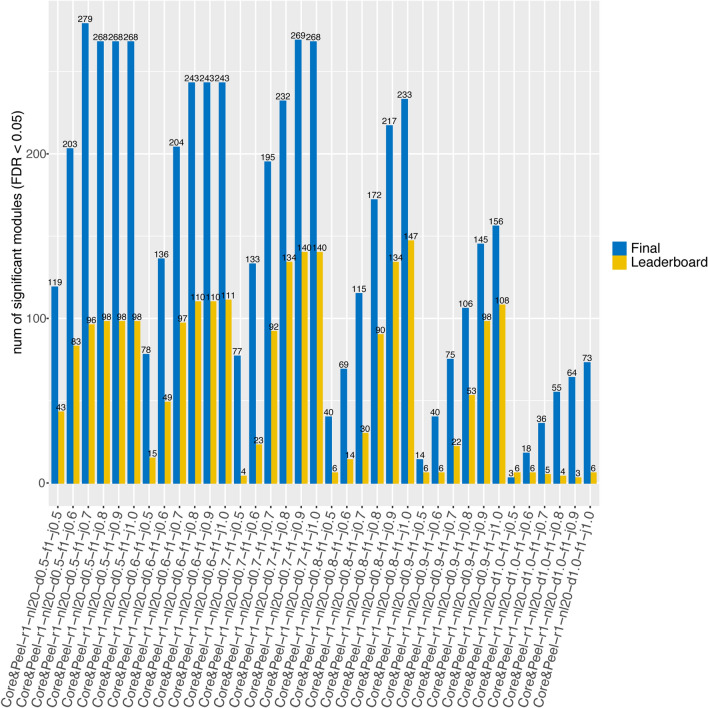
Number of disease-enriched modules detected by Core&Peel using the Leaderboard and Final GWAS datasets. The number of enriched modules were calculated by the Pascal tool using a 0.05 FDR cutoff. In abscissa the tested configurations of parameters for Core&Peel. We used the R package *ggplot2*^97^ to make the figure.

The DREAM challenge proposed the problem of identifying disease modules from six heterogeneous networks of proteins/genes. To test the performance of Core&Peel in detecting disease modules, we decided to use only the DREAM challenge gene co-expression network. We varied the Core&Peel parameters for subgraph density and Jaccard coefficient to find optimal settings (Fig. S1). We noticed that some modules had more than 100 genes. We removed these modules to be consistent with the DREAM challenge, requiring no module to have more than 100 genes (Fig. S2). The scoring methodology for the DREAM challenge used GWAS datasets as an orthogonal validation for the submitted networks. The PASCAL tool was used to correct the GWAS datasets for underlying linkage, creating gene-fusion regions tagged by the most significant SNP. By supplying PASCAL with the GWAS data and a gene network, this tool can score whether the detected modules contain significant GWAS hits. We tested the different Core&Peel parameters to the DREAM leaderboard (parameter tuning) and final submission GWAS datasets (Fig. 2). We chose the 0.7 density and 0.8 Jaccard index (more precisely Core&Peel-r1-nl20-d0.7-f1-j0.8 configuration) since it detected the largest number of enriched modules compared to other configurations in the leaderboard GWAS dataset. Generally when the Jaccard index is from 0.8 and 1.0 there is not a substantial variation in the number of disease modules identified (Fig. 2), so we decided to choose the smallest Jaccard index into this range. However when the density is too high (mostly equal to 1) very few modules were identified. This is more evident in the leaderboard step, in fact when the final GWAS datasets were used a remarkable improvement can be noticed, probably due to the larger number of final GWAS datasets than those in the leaderboard. We further noted that removing modules with a size greater than 100 slightly improves the performance (comparing Fig. 2 with Fig. S5).

#### Comparison of Core&Peel with DREAM methods

**Figure 3:**
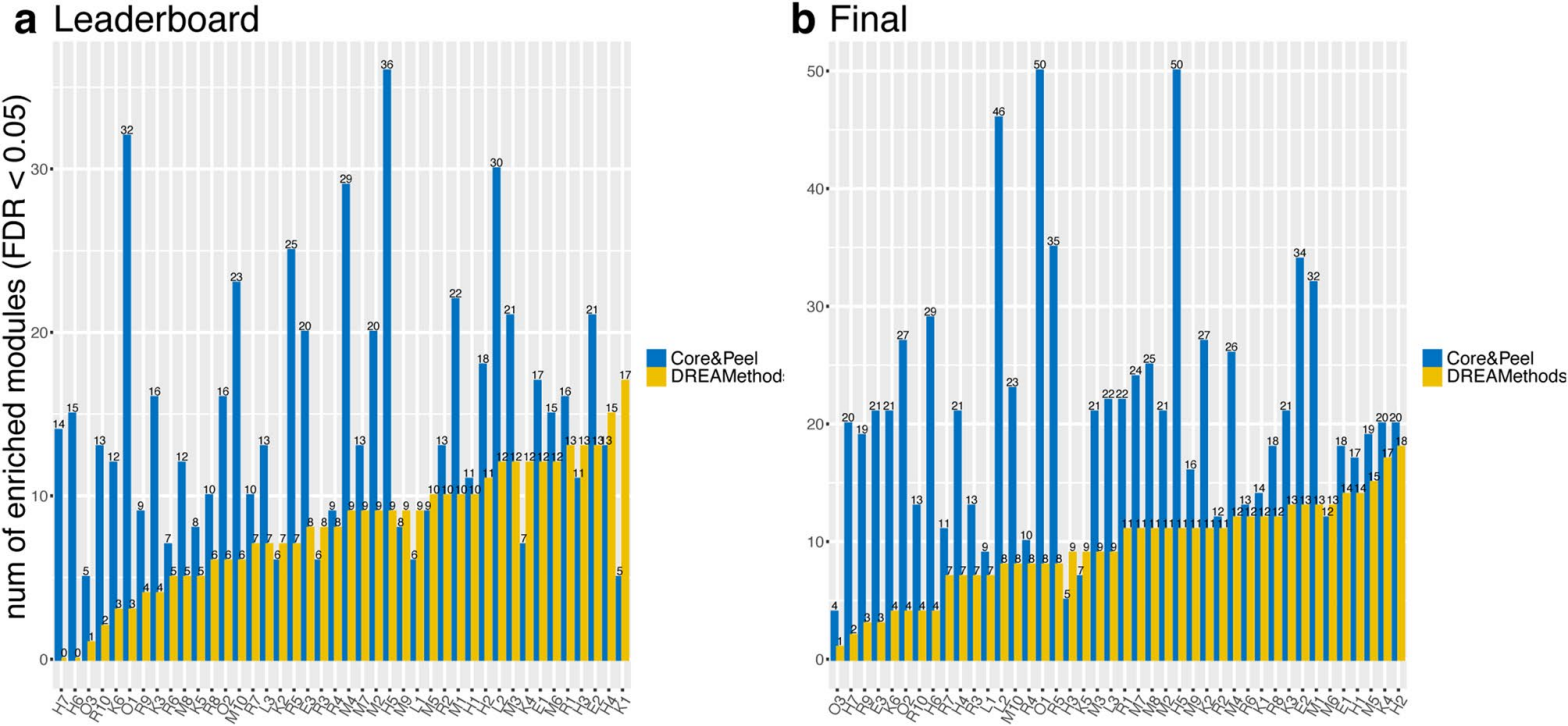
Comparison of number of disease modules between Core&Peel-r1-nl20-d0.7-f1-j0.8 and all the DREAM methods using Leaderboard (**a**) and Final (**b**) GWAS datasets. The number of enriched modules were calculated by the Pascal tool using a 0.05 FDR cutoff. The CRank ranking was applied to Core&Peel modules in order to select the same number of modules detected by each DREAM method. The x-axis is in ascending order respect to the number of enriched modules detected by DREAM methods. We used the R package *ggplot2*^97^ to make the figure.

The DREAM comparison methodology is fit for comparing methods producing non-overlapping modules, while we allow overlaps; we thus resort to introducing an explicit post-processing equalizing phase for Core&Peel (based on ranking and cut-off) when we compare the score of a method producing overlapping modules (Core&Peel) with a method producing non-overlapping modules. This is done for the sake of fairness of the comparison without sacrificing the advantages that overlapping modules have in modelling the underlying biology. In order to compare Core&Peel with DREAM methods, we thus applied the CRank ranking algorithm to the results of the configuration chosen (Core&Peel-r1-nl20-d0.7-f1-j0.8). We selected the highest rank communities to obtain the same number of modules of DREAM methods (Supplementary Fig. S3). The results of the leaderboard and final tests for Core&Peel versus the DREAM methods are represented in Fig. 3. In most of the cases, Core&Peel can identify more enriched modules. In a few cases, Core&Peel finds fewer GWAS enriched modules, for example when compared with K5 (Fig. 3b), probably due to the low number of predicted modules (equal to 16). Obviously, Core&Peel still has the advantage to have overlapping modules and this helps to obtain more disease enriched modules for an equal number of predicted modules with DREAM methods. This last aspect highlights the importance of taking into account modules with genes in common since they are more realistic from a biological point of view, as also demonstrated in^23^.

Moreover, we performed the GO analysis to evaluate the biological coherence of each predicted module. As before, we applied the CRank ranking to Core&Peel before conducting the enrichment analysis. We compared the number of predicted modules with different thresholds of significance (Supplementary Table S1), and Core&Peel enables us to detect on average more functional modules than DREAM methods, in particular when the FDR threshold is shallow.

**Figure 4:**
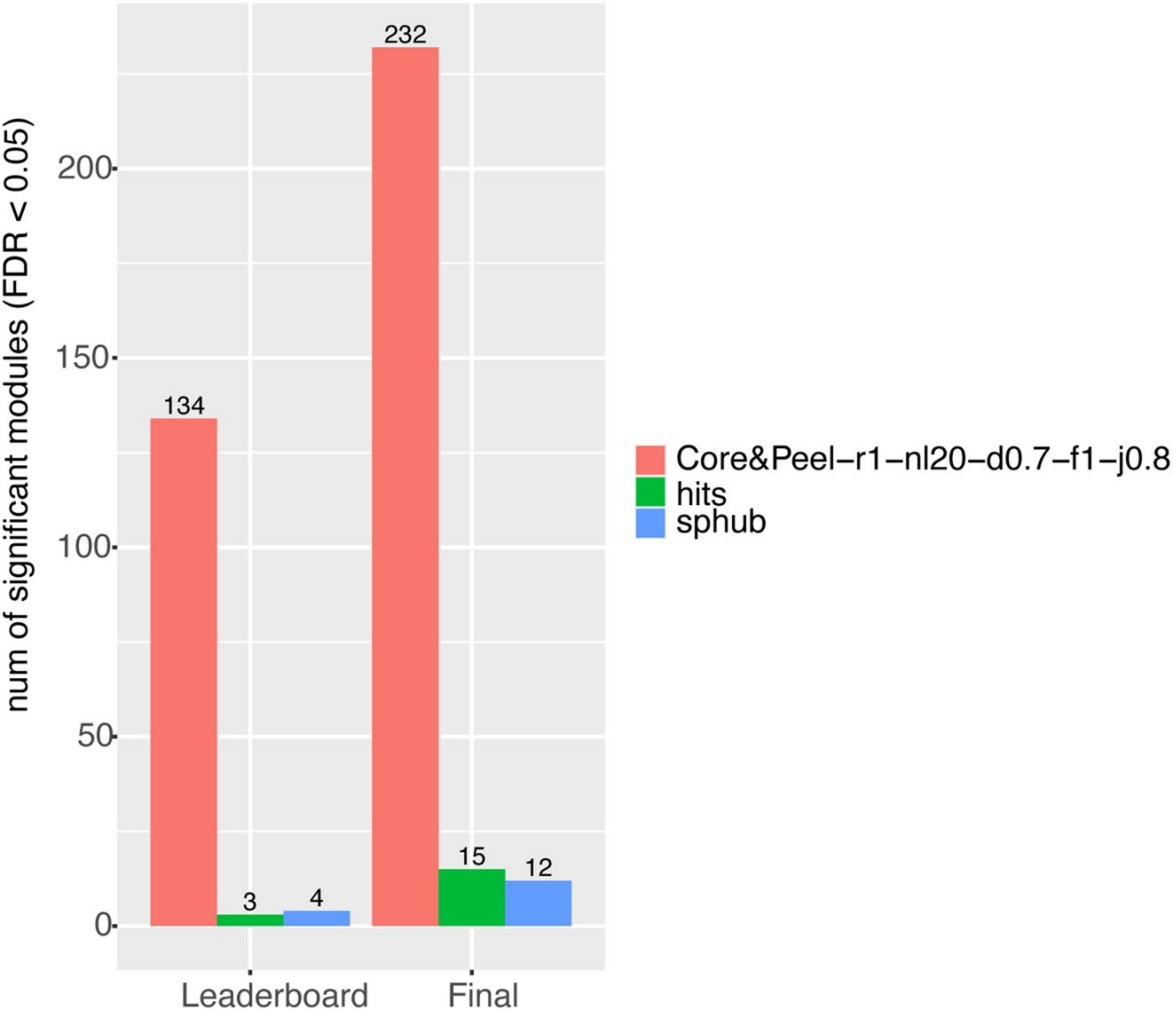
Number of disease enriched modules using both Leaderboard and Final GWAS datasets and comparing Core&Peel-r1-nl20-d0.7-f1-j0.8 with the two options (hits and spread hubs) of the method by Tripathi et al., explained in Section “A further method: overlapping community detection”. The number of enriched modules were calculated by the Pascal tool using a 0.05 FDR cutoff. We used the R package *ggplot2*^97^ to make the figure.

#### Comparison between Core&Peel and the overlapping community detection method

We compared Core&Peel with the method by Tripathi et al.^23^ described in the Section “A further method: overlapping community detection”, which also detects overlapping modules (i.e. a gene can belong to more than one module) like Core&Peel. Core&Peel predicts in total many more modules than this method (Supplementary Fig. S4), moreover Core&Peel identifies many more disease-enriched modules (Fig. 4). We also report the fraction of predicted disease modules (i.e. number of enriched modules divided by the total number of detected modules) in Table 1. Core&Peel identifies more disease modules both in absolute and relative terms, so we can consider it a competitive algorithm in disease modules identification problem, also for the class of methods producing overlapping modules. In Table 1 we reported the fraction of disease modules detected by the hits and spread hubs algorithms after filtering them according to the same Jaccard index (0.8) used in Core&Peel. We added this comparison to assess if there are any changes in the results when the rate of the overlap is the same among the two methods. However, we obtained similar outcomes with or without the Jaccard filtering. Besides, we analyzed the biological relevance of the predicted modules with GO analysis and Core&Peel achieves the best result (Supplementary Tables S2 and S3).

**Table 1:** Fraction of predicted disease modules (i.e. number of enriched modules over the total number of detected modules) that are enriched in the Leaderboard and Final GWAS datasets in Core&Peel-r1-nl20-d0.7-f1-j0.8 and in the two options (hits and spread_hubs) of the method explained in Section “A further method: overlapping community detection”.

	Core&Peel-r1-nl20-d0.7-f1-j0.8	hits	spread_hubs	hits_j0.8	spread_hubs_j0.8
Leaderboard	**0.021**	0.002	0.003	0.004	0.005
Final	**0.037**	0.01	0.01	0.006	0.01

The hits_j0.8 and spread_hubs_j0.8 indicate the original algorithms after filtering the modules according to the 0.8 Jaccard index. The hits and spread_hubs indicate the original algorithm without any filtering. The numbers in bold highlight the best value.

### Core&Peel: detection of active subnetworks

We next examined the performance of Core&Peel for detecting active subnetworks, i.e. finding subnetworks that are activated under different conditions. We used the DREAM challenge co-expression network and seven different datasets: two cancer RNA-Seq studies (prostate and colorectal cancer), two inflammatory disease microarray experiments (asthma and rheumatoid arthritis), and three COVID-19 related datasets (bronchioalveolar lavage fluid RNA-Seq, infected patient PBMCs, and COVID-19 infected A549 cells). We calculated differentially expressed genes using *edgeR* for the cancer data and *limma* for gene microarrays. We processed the first two COVID-19 data according to the pipeline the authors used; we used the summary statistics of the third dataset released from the authors to retrieve the differentially expressed genes (see Methods). We retained only modules that contained at least one differentially expressed gene in the comparison of interest. We ran Core&Peel using both nl=10 and nl=20 as the minimum subgraph size. We noticed that, in the active subnetwork detection problem, Core&Peel with nl = 10 identified more pathways with disease-associated genes than the case nl = 20. So we chose the Core&Peel-r1-nl10-d0.7-f1-j0.8 since it is one of the best configurations (same density and Jaccard coefficient) on the DREAM challenge data. Actually, this configuration works better in prostate and colorectal cancer cases where the number of differentially expressed genes (DEGs) is pretty high, instead when the number of DEGs is low (Supplementary Table S4), Core&Peel with a smaller density obtained better results. So we decided to use Core&Peel-r1-nl10-d0.5-f1-j0.8 in asthma and rheumatoid arthritis cases. In COVID-19 case studies, we took into account both Core&Peel configurations.

Core&Peel detected 1270, 141, 49 and 1495 modules in prostate cancer, asthma, rheumatoid arthritis, and colorectal cancer datasets, respectively. In COVID-19 cases, Core&Peel with density 0.5 detected 715, 558, and 109 in BALF, PBMC, and COVID-cells, respectively. Instead, Core&Peel with density 0.7 detected 545, 427, and 90 significant modules. As expected, the more are the DEGs (Supplementary Table S4), the more modules are identified. Since the significant modules were merged in one active subnetwork, we checked if this subnetwork is still significantly enriched with the DEGs; all active subnetworks produced got a p value $< 10^{-18}$ (data not shown).

**Table 2:** Number of genes in each active subnetwork detected by all the methods for the prostate and colorectal cancer, asthma and rheumatoid arthritis studies.

	Prostate	Asthma	Colorectal	Rheumatoid arthritis
Core&Peel	3048	1305	3883	519
MD	2708	192	2606	403
KPM	1266	74	2965	20
ClustEx	3000	1300	3800	500
Degas	122	37	44	85

Core&Peel-r1-nl10-d0.7-f1-j0.8 configuration was used for prostate and colorectal cancer, instead Core&Peel-r1-nl10-d0.5-f1-j0.8 for asthma and rheumatoid arthritis.

#### Comparison with other active subnetwork detection methods

We compared the Core&Peel modules with those detected by the four existing active subnetwork identification methods, namely ModuleDiscoverer (MD)^24^, KeyPathwayMiner (KPM)^25^, ClustEx^26^ and Degas^27^ (see description on Supplementary Notes Sect. 3.1). The number of genes in each active subnetwork is reported in Table 2. Generally, regulatory modules of asthma and rheumatoid arthritis include fewer genes than the prostate and colorectal cancer modules; this could be due to the lower number of DEGs. Degas is the method that produced the smallest modules. Core&Peel, ModuleDiscoverer and ClustEx produced the biggest ones, instead. Next, we were interested in comparing the modules detected by the different methods from a biological point of view. We compared the enriched pathways (Reactome-based) and plotted the overlap among all the method combinations (Fig. S6). We also conducted as baseline the pathway enrichment analysis using the DEGs. Globally, MD, ClustEx and Core&Peel detected a higher number of pathways than Degas, KPM, and DEGs. Degas is the method that identified the lowest number of pathways. In particular, in prostate and colorectal cancer cases (Fig. S6a,b), it does not overlap with the other methods. In both cancer cases, Core&Peel and MD share a significant part of their pathways (around 40) and a large number of pathways was identified only by ClustEx and MD in the prostate (Fig. S6a) and colorectal cancer (Fig. S6b), respectively. In the rheumatoid arthritis case, more than half of the pathways were shared by Core&Peel, ClustEx, and MD (Fig. S6d) and there is a small overlap for the other combinations. Overall a smaller number of enriched pathways were detected in asthma (see horizontal bars on Fig. S6c) than the other three case studies (horizontal bars on Fig. S6a,b,d) and we can also notice there are fewer overlapping pathways among the methods (Fig. S6c). Almost the entire set of enriched pathways of ClustEx is not in common with those produced by any other method. Similarly, more than half of the pathways identified by Core&Peel were not detected by the competing methods (Fig. S6c).

**Figure 5:**
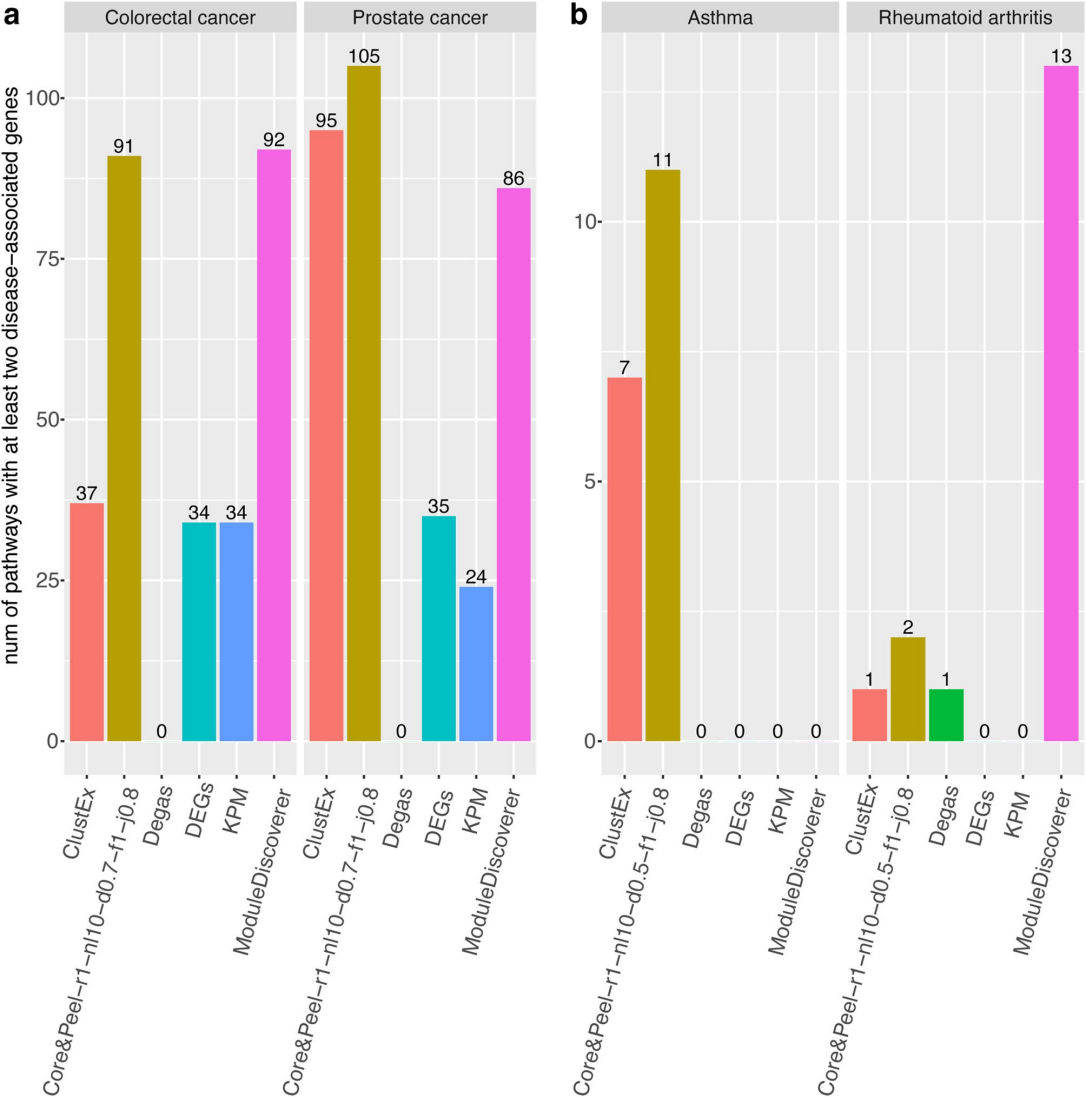
Number of enriched pathways with at least two disease-associated genes. The pathway enrichment analysis was performed for each subnetwork detected by Core&Peel and by the four competitive methods (ClustEx, Degas, KPM and ModuleDiscoverer) using the Reactome database. The pathway analysis using the differentially expressed genes was conducted as baseline. Pathways with an adjusted p value $< 0.05$ were selected and the number of disease-associated genes in each enriched pathway was calculated using the DisGeNET database. (**a**) Prostate and Colorectal cancer cases; Core&Peel-r1-nl10-d0.7-f1-j0.8 was used. (**b**) Asthma and rheumatoid arthritis cases where Core&Peel-r1-nl10-d0.5-f1-j0.8 configuration was used. We used the R package *ggplot2*^97^ to make the figure.

#### Detection of disease-associated pathways

To assess the performance in detecting pathways with genes associated with the disease, we calculated the number of enriched pathways with at least two genes associated with the specific disease (annotated on DisGeNET database). The results are shown in Fig. 5. Generally, Core&Peel can identify a substantial number of pathways. It indicates to be a competitive method, and it outperforms most of the algorithms. In particular, it performs exceptionally well in asthma case, in which most of the competitors can not detect any pathway with disease-associated genes. Core&Peel and ClustEx are the only methods in able to identify some of them, with Core&Peel catching a larger number of pathways (Fig. 5b). Overall more pathways consistent with disease-associated genes were detected in the two cancer datasets (Fig. 5a) than the asthma and rheumatoid arthritis datasets (Fig.  5b). This finding is consistent with the larger number of genes annotated on DisGeNET in cancer than asthma and rheumatoid arthritis. Generally Degas finds the fewest disease-associated pathways in all the case studies (almost zero pathways with disease-associated genes in all cases). Core&Peel, along with ClustEx and MD, can identify more pathways with disease-associated genes than DEGs; this highlights how the combination of network analysis and gene expression data can increase the power to detect pathways associated to the disease, which otherwise would not have been identified.

**Figure 6:**
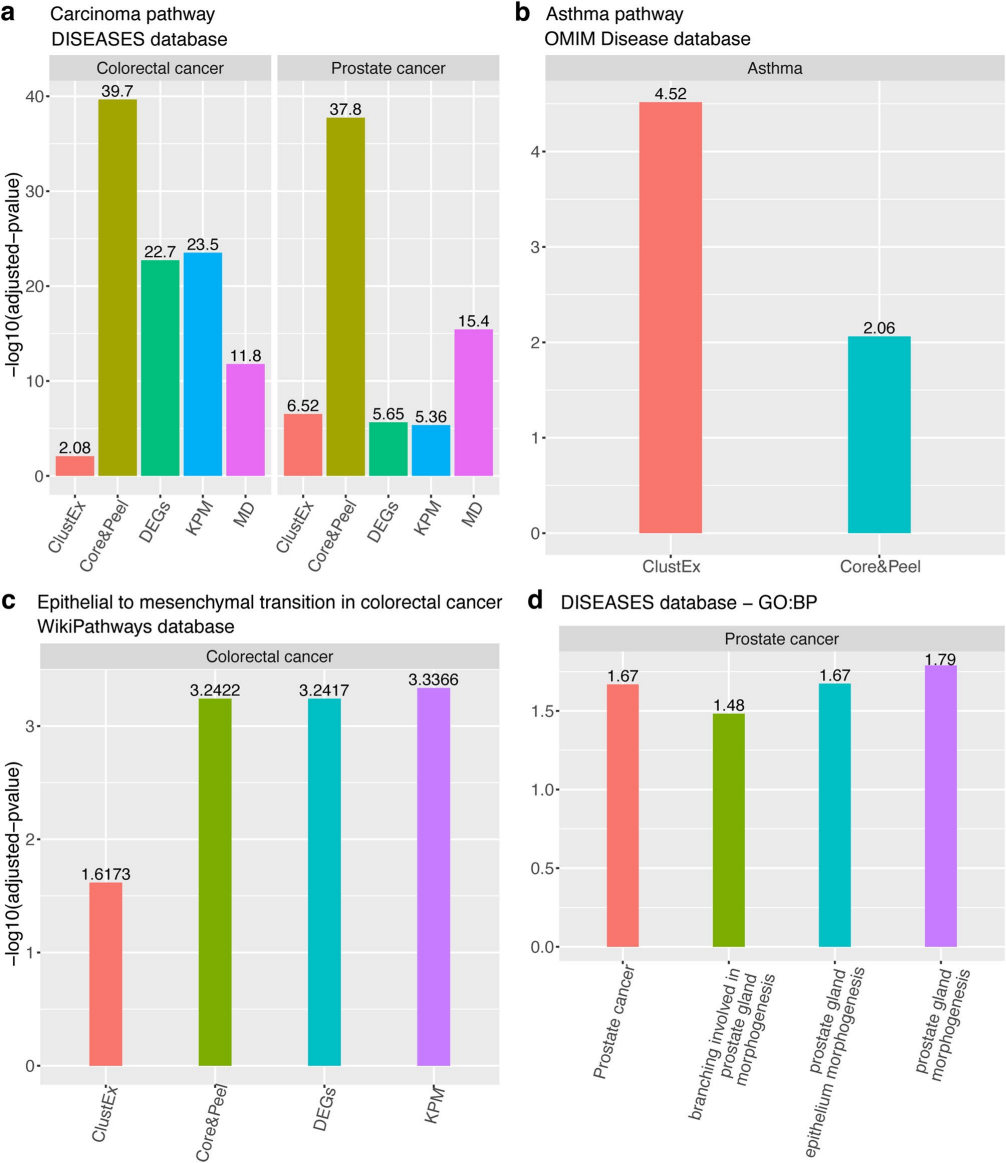
Results of *gProfileR* and *enrichR* enrichment analyses. The bars represent the adjusted p value in log10-scale. Only the methods which reached a significant p value ($<0.05$) are showed. (**a**) The adjusted p-values of Carcinoma pathway annotated in DISEASES database in colorectal and prostate cancer. (**b**) Adjusted p-values of asthma pathway annotated on OMIM_Disease database. (**c**) Adjusted p-values of epithelial to mesenchymal transition in colorectal cancer pathway annotated on WikiPathways database. (**d**) Adjusted p-values of Prostate cancer pathway (annotated on DISEASES database) and prostate gland morphogenesis, prostate gland epithelium morphogenesis and branching involved in prostate gland morphogenesis (annotated on GO). These pathways are enriched only in the Core&Peel active subnetwork. We used the R package *ggplot2*^97^ to make the figure.

Finally, we conducted further enrichment analyses using different databases to investigate the enrichment with specific-disease pathways. The results are shown in Fig. 6. Modules identified by Core&Peel, KPM, ClustEx, and MD (along with DEGs) are significantly enriched for the *Carcinoma* pathway in both cancer cases (Fig. 6a), suggesting their relevance to cancer in general. More specifically, the Core&Peel module is the only one to be significantly enriched for *prostate cancer* specific pathway (Fig. 6d). Moreover, also pathways involved in the prostate morphogenesis (*prostate gland morphogenesis, prostate gland epithelium morphogenesis* and *branching involved in prostate gland morphogenesis*) are significantly enriched and each of them contains some prostate cancer-associated genes, suggesting a possible involvement of these pathways in the prostate cancer (Fig. 6d). The *epithelial to mesenchymal transition in colorectal cancer* pathway is significantly enriched in Core&Peel module (Fig. 6c), giving a colorectal cancer specificity to the Core&Peel module. Moreover, the Core&Peel showed enrichment for the asthma pathway even if slightly less significant than ClustEx (Fig. 6b). No significantly enriched pathways were found for the rheumatoid arthritis case. Finally, in Supplementary notes (Sect. 3.2.2) we reported the same analysis using Core&Peel-r1-nl20-d0.8-f1-j0.8. Also in this case, Core&Peel can detect modules enriched for specific prostate and colorectal cancer pathways (*neoplasm of the colon, abnormal prostate morphology, prostate cancer* and *prostate neoplasm*) along with *Integrated Cancer Pathway* in colorectal cancer case. Besides, we analyzed the Reactome-based pathways detected only by Core&Peel for cancer along with asthma and rheumatoid arthritis cases. This analysis highlighted some significant pathways (with annotation in the literature), which could affect the studied diseases (see “ClueGO” section in Supplementary Material). Overall, these analyses have highlighted how Core&Peel can find modules specific to the disease.

#### Comparison with other active subnetwork detection methods on COVID-19 data

The numbers of genes in each module detected by all methods are reported in Supplementary Table S5. Both Core&Peel configurations (Core&Peel-r1-nl10-d0.5-f1-j0.8 and Core&Peel-r1-nl10-d0.7-f1-j0.8) have been tested to have a broader exploration of the enriched pathways. Consequently, two versions of ClustEx have been generated to get modules with size comparable with Core&Peel. In the three COVID-19 cases, the biological similarities between methods are different case by case. In BALF case (Supplementary Fig. S7), Degas has detected many pathways more than the other methods, and most of them are not in common among other methods. Core&Peel and ClustEx share a large part of the pathways. In the PBMC case (Supplementary Fig. S8), many pathways were detected only by ClustEx, and a large number of pathways are in common among Core&Peel, MD, and Degas. Finally, in COVID-19-infected cells case (Supplementary Fig. S9), Core&Peel detected the highest number of pathways, and just a few are in common with the other methods.

### Analysis of cellular transcriptional response to SARS-CoV-2 infection

Current research on network-based analysis of COVID-19 focuses mainly in analyzing host-pathogen protein interaction maps^18,19,28^ in order to find druggable target interactions. A second approach by Gysi et al.^17^ uses host-pathogens protein interaction to extract a subnetwork of a large human PPI network, which is further analyzed for possible drug targets. Here we change focus, analyzing the modularity and functional enrichment of active subnetworks in the host’s response to the infection, within a global host co-expression network. We applied active subnetwork detection methodologies to three case/control differential expression data sets on the human response to COVID-19 versus corresponding normal tissues/cell lines, using several active subnetwork detection algorithms. The complete list of significant GO BP functional annotations enriched in such active subnetworks is available at https://github.com/martaluc/CorePeel_activeSubnetwork. Next in this section, we comment on some of these annotations.

#### Broad biological processes

The first group of annotations comprises known pathways that are activated in viral infections, as well as in cellular apoptotic processes. Blanco-Melo et al.^29^ using DEA analysis noted that infection by COVID-19 has a molecular fingerprint characterized by “exuberant inflammatory cytokine production as a defining and driving feature of COVID-19”, and this is also noted by Xiong et al.^30^. Our analysis of active subnetworks confirms this finding at the level of enriched pathways in the active subnetworks: *positive regulation of cytokine production involved in inflammatory response* (by Core&Peel), *regulation of cytokine production involved in inflammatory response* (by Core&Peel), *cytokine production involved in inflammatory response* (by Core&Peel), and *Cytokines and Inflammatory Response WP530* (by Core&Peel and ClustEx) in Table S8. Pathway *Cytokines and Inflammatory Response WP530* is found enriched also in Supplementary Tables S6 and S7. Blanco-Melo et al.^29^ also noted a high expression of IL-6 as part of the characteristic COVID-19 signature. The importance of the interleukin activation is also shown in our analysis, as the pathway *IL-10 Anti-inflammatory Signaling Pathway WP4495*, which has regulatory effects on IL-6, is found enriched in Supplementary Tables S6 and S7, by ClustEx, and in Supplementary Table S8, by Core&Peel. Consistently with known mechanisms involved in SARS-CoV-2 progression^31^, we have found several apoptosis-related and lymphocite-related pathways in all three data sets. These observations are meant as a sanity check; their purpose is to recover in the functional enrichment analysis biological processes that are somewhat expected in any viral infection. These pathways *per se* are however usually aspecific and too broad to give useful hints for drug re-purposing efforts.

#### Specific biological processes hinting at candidate drugs for repositioning

Next we turn to discuss specific pathways for which it is more likely that existing drugs may alter their behaviour and have potential therapeutic effects. We keep our discussion at a pre-clinical level since the best we can hope from using the active network analysis is to find supporting evidence suggesting that some drugs (or families of drugs) should be shortlisted for further in vitro and *in vivo* experimental analysis, or finding additional evidence supporting drugs already shortlisted for clinical trials. Other sources and experiments may reasonably contradict the type of evidence we can hope to provide, therefore all available evidence should always be weighted in before moving to the next steps of a drug repurposing pipeline.

Functional annotations relative to Ebola virus (EBOV) infection is found enriched in Supplementary Table S8 (by all seven methods), S6 and S7, with strong p-values. This finding is of interest as it supports current attempts of repositioning drugs developed to cure Ebola infections in patients affected by COVID-19. One of the few drugs known to be acting on Ebola (at a pre-clinical level) is *remdesivir* (https://it.wikipedia.org/wiki/Remdesivir). Historical accounts of the development of the drug remdesivir^32,33^ show that interest on this drug in pre-clinical COVID-19 studies stemmed from its *in vitro* capability of inhibiting COVID-19/SARS-CoV2 infection^34^, and its known safety profile obtained in clinical trials for EBOV in 2014^35^. At the time of the study of Wang et al.^34^ remdesivir was already known, since 2017, to be effective *in vitro* and *in vivo* experiments involving members of the coronavirus family of viruses in a study by Sheahan et al.^36^, and this might be one of the reasons for shortlisting this drug in 2020^34^, along with other candidate drugs. Interestingly, our study of the patient’s transcriptional response to COVID-19 detects a direct link between the human response to EBOV and the response to COVID-19, at a modular level. In a hypothetical scenario in which one wishes to shortlist candidate drugs for an essay similar to Wang et al.^34^, our techniques would provide evidence that complements the supporting evidence provided by an essay identical to that in Sheahan et al.^36^.

A second set of enriched functional modules is HIV-related (annotations in Supplementary Tables S6, S7, and S8). This observation is consistent with the efforts in repositioning of AIDS therapeutic agents for COVID-19^37^. The NIH website (https://aidsinfo.nih.gov/understanding-hiv-aids/fact-sheets/21/58/fda-approved-hiv-medicines) lists about 23 FDA approved drugs effective in HIV/AIDS therapy (excluding combinations). One of these, *lopinair*, was found by in vitro experiments to be active against SARS^38^ and the Middle East respiratory syndrome coronavirus (MERS-CoV)^39,40^. Probably, for this reason, it has been selected for in vitro testing on cells infected by SARS-CoV2^41^. As in the previous case of the Ebola enriched module, in a hypothetical scenario, finding such HIV-enriched modules in the human transcriptional response to COVID-19 in late 2019/early 2020 would further support the choice of testing lopinavir by Cao et al. in early 2020. A few more drugs (or pre-drugs) effective in HIV therapy have been tested by Jockusch et al.^42^ and demonstrate potency in inhibiting SARS-CoV2 viral replication in cell cultures.

The *Staphylococcus aureus infection*, and *T-Cell antigen Receptor (TCR) pathway during Staphylococcus aureus infection WP3863* pathways are detected in Supplementary Tables S6, S7 and S8; while *Staphylococcus aureus infection* is detected in Table S6. Staphylococcus aureus is known to produce pulmonary infections^43^ thus it is a reasonable hypothesis that human response to *S.aureus* could share similarities at a molecular level with the COVID-19 response. Moreover, since several strands of *S.aureus* have developed resistance to traditional antibiotics, a new generation of antibiotic drugs have been introduced recently to cope with it. A listing of antibiotic drugs under consideration for repurposing on COVID-19 is reported in https://en.wikipedia.org/wiki/COVID-19_drug_repurposing_research and lists five antibiotics, among these four: Teicoplanin, Oritavancin, Dalbavancin, and Monensin are effective against Methicillin-Resistant Staphylococcus Aureus (MRSA)^44–47^. Baron et al.^48^ shortlisted Teicoplanin in in vitro testing for activity against COVID-19. Oritavancin, Dalbavancin, and Monensin have been found to inhibit the entry of Ebola, MERS, and SARS viruses in in vitro tests^49^. Again, finding *S.aureus* enriched pathways in human transcriptomic response would indicate the opportunity for shortlisting modern antibiotics active in severe *S.aureus* infections for use in COVID-19 in vitro testing.

We could find the pathway *Inflammatory mediator regulation of TRP channels* enriched in Supplementary Tables S8 and S6. Transient receptor potential channels have been studied for their role in inflammatory processes and as therapeutic targets^50–53^. This observation supports another possible line of attack in the search for COVID-19 treatments^54,55^.

Two tables in supplementary material show enriched pathways for response to Cytomegalovirus (CMV). Jockusch et al.^42,56^ report that three drugs known to be active for AIDS-related cytomegalovirus (CMV) infections inhibit the SARS-CoV-2 RNA-dependent RNA polymerase (RdRp), and have potential to evade COVID-19 exonuclease activity. These drugs belong to a class of drugs known to inhibit SARS coronavirus infections in in vitro tests^57^. Thus also in this case, the detection of enriched pathways typical of the response to CMV and HIV in our analysis, would support shortlisting of the family of drugs selected in by Jockusch et al.^42,56^.

#### Other Specific biological processes

The third group of enriched pathways points at specific transcriptional response to pathogens. However, it is not clear whether indication of a candidate drug (or class of drugs) emerges at this stage. Enriched pathways for the response to Epstein-Barr virus (EBV) are found in Supplementary Tables S6, S7, and S8. Cases of patients co-infected by COVID-19 and EBV have been reported^58^, however, our active subnetwork analysis suggests that also these two viruses may provoke similar response profiles in patients at a molecular level. Other pathogen-response modules enriched in the active subnetworks detected in all three data sets (Supplementary Tables S6, S7, and S8) are relative to Escherichia coli and Human papillomavirus. Two tables in supplementary material show enriched pathways for the response to Herpesvirus, Helicobacter pylori, Salmonella, Yersinia, and Cholera. We cannot rule out that a case of co-infection by a variety of pathogens could explain some statistical associations found in the data^59^. This event is however, less likely for the data obtained through cellular lines rather than patient samples. A second possibility is that the systemic human responses to these pathogens are much overlapping among themselves, besides being enriched in COVID-19 data, thus the statistical associations may not be independent of each other.

Chloroquine (Hydroxychloroquine) was one of the first drugs to move into the clinical trial stage for COVID-19 rising much expectation and controversy (see e.g. Pastick et al.^60^, Hoffmann et al.^61^). Limiting the discussion to the pre-clinical level, several in vitro experiments on SARS and SARS-CoV2 showed activity of Chloroquine^34,62–65^. Chloroquine is mostly known as an anti-malaria drug, while in our data, we could not spot specific pathways related to malaria, and therefore no indication for shortlisting it. With hindsight, though, a very indirect link can be spotted by combining a rarely cited result of 1990^66^ that reports on Chloroquine’s activity of on Vibrio cholerae, and our finding of a cholera-related enriched pathway. Recent results^61^ show however, that the action of Chloroquine in in vitro experiments may be tissue-dependent, thus making it harder to generalize such findings.

## Discussion

In this work, we explore the potential value of the Core&Peel method for solving two related problems. The first problem is functional module detection within a biological co-expression (co-expr) network of genes. The second is the problem of active functional module detection, that is, finding modules within a biological network of genes, that are most affected in a specific disease vs. a normal background state. We initially discuss the two problems separately as the validation methodology is different in either case, however, the two aspects of the issue often interact with each other.

Firstly, we compare Core&Peel with 42 methods producing non-overlapping modules that participated in the DREAM challenge project, as well as some recent methods of Tripathi et al. 2019, which allow substantial module overlap. Module overlap is an issue that often arises in the context of network partitions and modularization (as also in graph-based community detection). The DREAM challenge did require competing methods to report non-overlapping modules. This choice has the first effect since there is no need in the DREAM challenge of a requirement on the maximum number of produced modules. The number of nodes in a network is a natural upper bound on the number of reported modules. It is also somewhat easier to evaluate the enrichment of disjoint modules since one does not have to cope with inflated results, where possibly many high quality overlapping modules differ by just one or two genes, collectively representing quasi-duplicates of the same biological module. On the other hand, there is ample evidence^67–70^ (see also the discussion in the DREAM paper^22^) that functional biological modules often share sub-modules and that this sharing has precise implications for the biological interpretation of the processes under study. When we compare Core&Peel with the output of the DREAM challenge, we mitigate the potential bias in the measurements due to overlapping modules by imposing a maximum value of the Jaccard coefficient between any pair of modules, thus avoiding to report quasi-duplicates. Moreover, for the sake of comparison, we rank the modules by intrinsic topological quality (see^71^) and we limit Core&Peel to the top k modules in this ranking when we compare it to a method reporting k modules (for the same input graph).

Within these caveats, we strictly follow the performance measurement methodology of DREAM. We use the GWAS leaderboard and the final datasets separately (see Fig. 2). The results on the GWAS leaderboard are used to optimize the choice of the parameters of the Core&Peel method. Figure 2 reports good qualitative concordance of the relative performance of Core&Peel between leaderboard and final GWAS datasets for a wide range of parameters. There is a discrepancy for the density value $d=1.0$, which corresponds to detecting full cliques in the input graph. Since even the best co-expression networks are approximations to the true network of interactions, missing edges (false negatives) are expected in the input, thus implying that quasi-cliques may be more relevant to functional module detection than full-cliques.

Figure 3 reports the comparison of the absolute number of enriched modules found by Core&Peel (with the selected parameters) versus the DREAM methods. Both for the leaderboard and the final GWAS data, Core&Peel restricted to the top k modules finds more enriched modules most of the times (for final GWAS 39/42 times, for leaderboard GWAS 35/42 times). Comparing the method of Tripathi et al. 2019^23^, results in Table 1 and Fig. 4 show that Core&Peel can detect many more significant modules (with a common Jaccard coefficient maximum threshold of 0.8), and that Core&Peel has a larger fraction of the reported modules enriched for GWAS data. We also performed a second analysis in which we count the number of modules enriched within one (or more) Gene Ontology annotation at an FDR below a fixed threshold (see Supplementary Tables S1, S2, and S3). Supplementary Table S1 reports the number of enriched modules for the DREAM methods, and the average number overall DREAM methods. The average number of GO:BP enriched modules for the DREAM methods is comparable to those found by Core&Peel for thresholds $10^{-2}$, and $10^{-3}$, however, for thresholds from $10^{-4}$, down to $10^{-7}$ Core&Peel has a substantially higher number of enriched modules. At threshold $10^{-7}$ Core&Peel reports more enriched modules than 39/42 of the DREAM methods. Comparing the method of Tripathi et al.^23^, results in Supplementary Table S2 and S3 show superior performance of Core&Peel, both in terms of enriched modules for GO categories, and in terms of the fraction of enriched modules over the total number of returned modules. Both the validation with GWAS data and that with GO data give the same picture, showing that Core&Peel is competitive with the best methods in the field on many measured quality functions, even when discounting for the difference in the number of reported modules. Moreover as noted in^72^ (page 8) the validation via GWAS enrichment uses data derived from association studies that are not normally used to define pathways and functional categories found in databases.

Supplementary Tables S2 and S3 show that at threshold $10^{-4}$ Core&Peel reports 6285 modules, and more than 53% of these (3343) are enriched with one or more GO annotations. This is a very rich structure, and we can exploit this richness to attack the problem of active module detection. Similarly to other methods, like ModuleDiscoverer, we use the list of differentially expressed genes (DEGs) in case-control experiments to compute the enrichment of each module in DEGs, and we report as active modules those with an enrichment below a given FDR threshold (typically 0.01). Other methods like Degas^27^ (which is available the suite Matisse^73^) take a different approach, as the original input graph is annotated with DEGs, and the subnetwork induced by the DEG nodes is used as a seed to discover a minimally connected subgraph that joins all DEGs.

The main validation methodology for the active subnetwork detection methods involves finding validated disease-related pathways and testing the enrichment of such pathways in the active subnetworks. For this type of analysis (over the four test cases), we use several curated databases of pathways. The association of the pathway to the disease can be either obvious from the pathway textual description, or it can be inferred by the presence of disease-related genes (at least two). Figure 5a and b report the number of affected pathways, obtained by combining the Reactome and DisGeNET information, enriched in the predicted active subnetworks. We note that only two methods (Core&Peel and ClustEx) can detect significant pathways on all the four test cases. ModuleDiscoverer finds at least one pathway in three test cases, while DEGs and KPM find pathways in two test cases and Degas in only one. Although Core&Peel and ClustEx do report enriched pathways on all four datasets, Core&Peel does find more such pathways (and for prostate cancer, substantially more). Although any of the competing methods (ClustEx, KPM, ModuleDiscoverer, and Degas) may perform better than Core&Peel in some particular case, Core&Peel can perform uniformly well across all four test cases, and it is the only method in the comparative evaluation that could find significant enrichment for many validated cancer pathways.

All methods mentioned in this paper require in input a biological network in which nodes represent genes, and edges represent interactions between pairs of genes. Many active subnetwork detection methods have been developed and tested using as input PPI networks (protein-protein interaction networks)^5^. Instead for this study we chose as target (large) gene co-expression network developed within the DREAM challenge project. There are advantages and supporting evidence that using a co-expr network is a sensible choice. (a) an edge in co-expression network may be due both to direct protein-protein interactions of the proteins associated with the genes, as measured in large scale proteomic essays. However it may also be due to indirect co-regulation in which the two genes show correlation because their expression is modulated by a common Transcription Factor or an ncRNA (miRNA, lncRNA, etc..). Thus a richer array of phenomena is captured in a co-expr network. (b) Co-expr networks tend to have more edges and thus to be denser than most PPIN. This is an advantage for density based-methods, as modules tend to be more compact (in other words the average path length within a module is shorter for the same module detected in a denser network). (c) Co-expr networks are usually based on the correlation between expression vectors of pairs of genes, across thousands of measurements of gene panels (via microarray or RNAseq technologies). This set up integrates naturally data from different experiments, and different platforms, via standard normalization. The resulting correlation values depend very little on outlier conditions or variations across experimental conditions, thus representing a robust datum. In contrast, the integration of PPI data is somewhat more affected by outlier experiments and heterogeneous experimental conditions. (d) Huang et al.^74^ compare the performance of 21 networks in uncovering pathways associated with a particular disease phenotype, by using 50 literature-based validated pathways. Huang et al. note that larger network size gives a higher absolute performance score. Moreover, among seven networks integrating co-expression data, six rank in the top seven positions by absolute performance score. (e) The findings in^74^ are also consistent with the observations in^22^ as the largest average number of disease-related modules is detected (averaging among methods) in the co-expression network (of 1M edges). Slightly less performant is a PPI network which, however, has more than twice as many edges (about 2.2 M). On balance, although there is evidence pointing at co-expression networks as an input of choice, both^22^ and^74^ indicate that usually, different types of networks reveal various aspects of the active subnetworks. Thus a multi-facet approach should always be considered for specific investigations.

In our active subnetwork detection approach, we have as input both a biological gene interaction network (called the “bio-network” data) and a list of gene expression data on cohorts of cases and controls for a disease under study (called the “perturbation” data). The bio-network represents a global and essentially static picture of the cell’s machinery, while the perturbation represents the dynamic element we wish to study within the context of the bio-network. All the methods we compare with imply these two roles (static/dynamic) for the two inputs (network/perturbation), although they may differ in many other aspects. A limitation of this view on the data is that the phenomenon of network rewiring in response to the stimulus is captured somewhat imperfectly. Rare novel gene interactions (i.e. new edges in the network) arising as the result of the stimulus are not included in this model, and cannot be predicted. There is a different family of approaches (see e.g.^75^) that focus instead on the network rewiring phenomenon, by a more direct experimental assessment of dynamics of physical and genetic networks, through experimental mapping of networks. In this setting typically one would produce and analyze two full networks, one representing the system’s status before and one after the stimulus, to capture the variations. Here there is a different trade-off between the complexity of the experimental setup and the depth of the downstream ’in silico’ analysis.

## Conclusion

In the first part of this research, we aim at comparing Core&Peel with competing methods on benchmark data. Adapting the DREAM challenge evaluation methodology to the case of overlapping functional modules we conclude that, in terms of the number of enriched detected modules, Core&Peel is competitive with 42 functional module detection methods (which report non-overlapping modules) participating in the DREAM Challenge, and with the recent method by Raman’s group^23^ (which allows overlapping modules). Naturally, the number of enriched modules detected is just one easy-to-measure parameter, and other features may be important in a comparative assessment of several methods for the same task. On a more qualitative level, comparison of Core&Peel with several active subnetwork detection algorithms on four benchmark test cases show that Core&Peel has more uniform performance across all these four benchmark datasets, and for two cancer data sets (prostate, and colorectal) it produces modules enriched with more specific pathways, overlooked by the competing methods. COVID-19 is a recent infectious disease caused by severe acute respiratory syndrome coronavirus 2 (SARS-CoV-2) first isolated in December 2019. As relatively little is known of this contagious disease at this time, we adopt an exploratory approach using Core&Peel along with several other methods to uncover active subnetworks of human response to COVID-19. Pathway enrichment analysis of such active subnetworks uncovers some broad biological processes common to many infections, as well as more specific pathways that show similarities between human transcriptional response to SARS-CoV-2 and response to other pathogens, and thus provide evidence useful in ongoing drug repositioning efforts at a pre-clinical level.

## Availability and usage

Core&Peel algorithm is available here http://bioalgo.iit.cnr.it/index.php?pg=ppin. The active subnetwork detection script is available as Docker image https://hub.docker.com/repository/docker/ma10r02t90a/corepeel_active-subnet/general (see also GitHub repository https://github.com/martaluc/CorePeel_activeSubnetwork). The Github repository includes a user manual to help users applying the pipeline to custom data.

## Methods

### DREAM challenge

The “Disease Module Identification DREAM challenge”^22,72^ is an open competition to assess disease-module identification methods across heterogeneous biological networks for homo sapiens. In this section, we summarize the data and the methods that were made available by the challenge. We mention a further recent method developed for the module detection problem, which uses data from the challenge by Tripathi et al. 2019^23^. We introduce the strategy used to test for the association of the predicted modules with complex traits and diseases using a collection of Genome-Wide Association Study (GWAS) datasets and a tool for module scoring called PASCAL^76^. Finally, we introduce the Gene Ontology (GO) analysis we performed to evaluate the biological coherence of each predicted module.

#### Co-expression network

Among the six different biological networks available from the DREAM challenge, we decided to focus our attention on the co-expression network. This network is based on Affymetrix HG-U133 Plus 2 arrays extracted from the Gene Expression Omnibus (GEO)^77^. A gene expression matrix of 22268 genes by 19019 samples was obtained, and the pairwise Spearman correlation of genes across samples was computed. The network was created with genes as nodes and the correlation coefficients as edge weights. In order to reduce the noise, we removed all the edges with a weight lower than 0.25, obtaining a network with 12477 nodes and 665187 edges.

#### DREAM methods

Forty-two methods took part in the DREAM challenge, and we compared our results with every algorithm (called DREAM methods in this manuscript). These methods are classified into different categories: (i) kernel clustering, (ii) modularity optimization, (iii) random-walk based, (iv) local methods, (v) ensemble clustering, (vi) hybrid methods and (vii) others. For more details see Table S1 in^22,72^. The common characteristic of DREAM methods is to identify non-overlapping modules.

#### A further method: overlapping community detection

Apart from comparing the Core&Peel results with DREAM ones, we took into account a further method developed by the Raman group^23^. This method has been designed to detect disease modules starting from heterogeneous biological networks. Like Core&Peel, it uses the data from the DREAM challenge and can detect overlapping modules. Basically this method involves two steps: (i) seed nodes selection and (ii) seed expansion. The seed nodes selection relies on the observation that disease genes have a higher degree than the non-disease genes. Consequently, the authors used HITS and spread hubs, which are based on the degree of a node, as a seed selection mechanism to select some important nodes from the network. After that, for each seed node, they applied a seed expansion algorithm that uses the Personalized PageRank score to rank the nodes in the neighborhood of a seed node. The nodes were added to the module one by one based on their ranking and the modularity score was re-calculated after adding every node. The set of nodes with the maximum modularity formed a module.

We used their code (seed selection and seed expansion) released on GitHub: https://github.com/RamanLab/DiseaseModuleIdentification. We applied the same co-expression network used in Core&Peel as input data. The only tunable parameter of this method is the number of the seed nodes to set in the seed nodes selection script. The authors tested different values of this parameter (see ’Co-expression network’ Table S4 in the Supplementary Material of the original paper https://www.frontiersin.org/articles/10.3389/fgene.2019.00164/full#supplementary-material) but we chose the maximum one (4099) since the smaller the value, the fewer disease-enriched modules were detected. Using this value, we generated the maximum number of HITS and spread hubs seed nodes; in particular the algorithm found 1423 and 1279 HITS and spread hubs, respectively. Additionally we filtered the modules detected by this method according to the same Jaccard index used in Core&Peel to have the same rate of overlap between the two methods.

#### Genome-wide association study (GWAS)

A Genome-Wide Association Study (GWAS) is an approach used in genetics to identify genetic variants associated with the risk of disease or a particular trait. In particular, this method searches the genome for small variations, called Single Nucleotide Polymorphisms (SNPs), which consist of a single nucleotide alteration. Single SNPs usually do not have large effects on disease risk of an individual, but they can be associated with increased risk at population scale. The DREAM challenge provides a collection of 180 GWAS datasets from public sources. This collection was split into two sets of 76 and 104 GWASs used for the leaderboard step (parameter optimization) and the final evaluation, respectively. We used both sets separately to test the performance of the methods.

#### Gene and module scoring using Pascal

PASCAL^76^ (PAthway SCoring ALgorithm) is a powerful tool for computing gene and pathway scores from SNP-phenotype association p-values. Pascal uses analytic and numerical solutions to calculate gene and module scores from the SNP p-values correcting for linkage disequilibrium (LD) correlation structure. PASCAL was used to get a score for each method. This score was defined as the number of modules with significant Pascal p-values in at least one GWAS (if a module is significant for multiple GWAS traits, it was counted once). The Pascal p-values were adjusted to control the FDR via the Benjamini-Hochberg procedure, and a 5% FDR cutoff was applied. This methodology is also used in^78^.

#### Gene ontology (GO) analysis

To assess the biological coherence and relevance of each predicted module, we performed the GO analysis. Basically, we computed the hypergeometric p-value of the association of each module to each GO class. We used the *hypergeom* method implemented in the *scipy.stats* package of Python. For each module, we assigned the GO class of lowest p value. In order to correct for multiple comparisons, we adjusted the p-values using the *p.adjust* R function with *fdr* method which is the Benjamini-Hochberg FDR estimation method. The GO database was downloaded by ftp://ftp.ebi.ac.uk/pub/databases/GO/goa/HUMAN/goa_human.gaf.gz and the Biological Processes (BP) category was selected. Additionally, only the genes in the co-expression network were retained as background.

### Core&Peel

In this section we briefly summarize the algorithm’s main features; for more details refer to^21^. Basically, Core&Peel aims at identifying dense subgraphs from large or medium size networks, which are both ego-networks and quasi-cliques. Briefly, it constructs a set of neighbors for each node of the graph based on its core number. Afterward it applies a peeling step that iteratively removes nodes of minimum degree in the graph. The peeling procedure stops when the number of nodes drops below the minimum size threshold or when the density is above or equal to the minimum density threshold. Finally, the duplicates were removed, and if two subgraphs were too similar according to the Jaccard coefficient, the biggest one was retained.

For both our purposes, we assessed the Core&Peel performance, optimizing different control parameters. The subgraph minimum density (d) and the maximum Jaccard coefficient (j) varied from 0.5 to 1.0, and the minimum subgraph size (nl) was set to 10 or 20. Instead, the filter strategy (f) was fixed to 1 because it is neither too strict nor loose and the distance from the seed node (r) was set to 1.

#### CRank

CRank^71^ is a general approach for prioritizing network communities. It takes a network and detected communities as its input and produces a ranked list of communities, where the high-ranking ones represent the promising candidates for downstream analyses. It is based on the evaluation of four different structural features of each community: (i) the likelihood of the edges, (ii) internal connectivity, (iii) external connectivity, and (iv) relationship with the rest of the network. CRank then applies a rank aggregation method to combine these measures in order to produce the final ranking list of communities.

We applied this ranking method on the communities detected by Core&Peel to compare them with the modules detected by the algorithms of the DREAM challenge. This aimed at reducing the effect of the overlap of the Core&Peel communities when compared with DREAM method communities, which do not contain any common genes between them.

Basically, we selected the first k-communities of Core&Peel with the highest CRank scores, where k is the number of communities detected by the DREAM method we want to compare our result with. In other words, Core&Peel results were compared with every DREAM method, taking the same number of communities (in most of the cases, Core&Peel detects more communities than DREAM methods).

#### Selection of active subnetworks

The second purpose of this work is to assess the Core&Peel performance in active subnetwork detection. We used the same Core&Peel modules calculated previously for the identification of disease modules. Among them we selected only the subnetworks that include genes that have a significant change of their expression in the patients with disease with respect to the healthy samples. More specifically, for each case study, we calculated the significance of the overlap between each module and the differentially expressed genes (DEGs) through the hypergeometric test. We took into account only the modules with a p value of less than or equal to 0.01. The p-values are computed by the *hypergeom* method implemented in the *scipy.stats* (version 1.1.0) package of Python (version 3.7). Finally, all the significant modules are merged in one, taking each gene once. We made this decision to facilitate the comparison of our results with the other methods, which did not take into account modules separately.

### Transcriptomic data, pre-processing and differential expression analysis

This section summarizes the datasets used to identify the active subnetworks from the co-expression network and gene expression data. In total, seven gene expression data have been analyzed, two are from The Cancer Genome Atlas (TCGA), two are from GEO, and the other three from COVID-19 studies. We also describe the differential expression analysis (DEA) we performed to get the DEGs, which were used for the active subnetworks selection (Table S4).

#### TCGA data

We downloaded and pre-processed the RNA-Seq data from TCGA for prostate and colorectal cancer using the *TCGAbiolinks* R/Bioconductor package^79–81^. We performed the DEA with the *edgeR* R package^82^. We compared the tumor with the normal samples, and a False Discovery Rate (FDR) cutoff was set to 0.01, along with log fold-change lower threshold $|log(FC)| \ge 1$.

#### GEO data

We downloaded the microarray data from GEO for asthma (GSE137268) and rheumatoid arthritis (GSE15573). The normalized data were downloaded using the *GEOquery* R package^83^. Differential expression analyses have been carried out with *limma*^84^ setting the FDR threshold to 0.05 and FC lower threshold $|log(FC)| \ge 1$. We decided to increase the FDR threshold respect to that one chosen for the TCGA data to obtain a higher number of DEGs.

#### COVID-19 data

We retrieved the DEGs from three COVID-19 datasets included in two different studies. In the first study, Xiong et al.^30^ carried out transcriptome sequencing of the RNAs isolated from the bronchoalveolar lavage fluid (BALF) and peripheral blood mononuclear cells (PBMCs) specimens of COVID-19 patients. In both cases, they compared the case with healthy patients, and we retrieved the DEGs using the pipeline released by authors. Instead, Blanco-Melo et al.^29^ infected the human adenocarcinomic alveolar basal epithelial (A549) cells with SARS-CoV-2 (COVID-19-cells case) and compared them with controls. The authors released only the statistical parameters (ex: logFC, adjusted p value, etc...) from the DEA for each gene. We selected the DEGs choosing the FDR less than 0.05 and $|log(FC)| \ge 1$. Additionally, the R DESeq package was used to perform DEA in all the three COVID-19 studies, but the authors released their data differently; in the first work the DEA code was available on GitHub (https://github.com/zhouyulab/ncov/tree/master/figs), in the second one only the summary statistics were made available (Supplementary Table S2 in https://www.biorxiv.org/content/10.1101/2020.03.24.004655v1.supplementary-material).

### Validation of the active subnetwork

In this section, we sum up some enrichment analyses conducted to evaluate each method’s power to detect pathways associated to the diseases studied in this work. The R software has been used in these analyses^85^.

#### Pathway enrichment analysis: reactome and DisGeNET

We performed the pathway enrichment analysis based on the Reactome database for each active subnetwork obtained for each case study. We employed the *enrichPathway* function of the R package *ReactomePA*^86^ to retrieve the enriched pathways with adjusted p-values below the 0.05 cutoff. We calculated the number of disease-associated genes in each enriched pathway using the DisGeNET database^87^. Lastly, we calculated the number of enriched pathways with at least two disease-associated genes.

#### Enrichment analyses based on different databases

Different databases including specific-disease pathways are available. In order to get a broader investigation of these pathways, we decided to use two R packages *enrichR*^88^ and *gprofiler2*^89^, which use different databases of interest. In the first case, we conducted the analysis using DISEASES^90^, OMIM_Disease^91^ and WikiPathways_2019_Human^92^ databases. In the second case, we took into account the KEGG^93^, GO^94^ (BP category) and Human phenotype ontology databases^95^. For each analysis we extracted enriched pathways (with adjusted p value < 0.05) whose description included specific key-words (example: “prostate”, “carcinoma”, “asthma”, “colon”, “rheumatoid”, “virus”, “infection”) and compared their adjusted p-values in -log10-scale.

## Supplementary information

Supplementary information.
