# Supplementary material for: Finding disease modules for cancer and COVID-19 in gene co-expression networks with the Core&Peel method
Source: Sci Rep. 2020 Oct 19;10:17628. doi: 10.1038/s41598-020-74705-6 (PMC7573595; doi:10.1038/s41598-020-74705-6)
Supplement: Supplementary file 1 — Supplementary information. [file 41598_2020_74705_MOESM1_ESM.pdf]

Finding disease modules for cancer and COVID-19 in gene co-expression networks with the Core&Peel method
# - supplementary materials -

Marta Lucchetta[1,2] and Marco Pellegrini[1,*]

[1]Institute of Informatics and Telematics (IIT), CNR, Pisa, 56124, Italy
[2]Department of Biotechnology, Chemistry and Pharmacy, University of Siena, Siena, 53100, Italy
[*]marco.pellegrini@iit.cnr.it

Contents

1 Supplementary Figures **2**

2 Supplementary Tables **11**

3 Supplementary Notes **17**
3.1 Algorithms for active subnetwork identification . . . 17
3.2 Core&Peel: detection of active sub-network when nl = 20 . . . 17
3.2.1 Comparison with other active sub-network detection methods . . . 18
3.2.2 Detection of disease-associated pathways . . . 18
3.3 ClueGO analysis . . . 22
3.3.1 Prostate cancer . . . 22
3.3.2 Colorectal cancer . . . 24
3.3.3 Rheumatoid arthritis (RA) . . . 26
3.3.4 Asthma . . . 27

1 Supplementary Figures

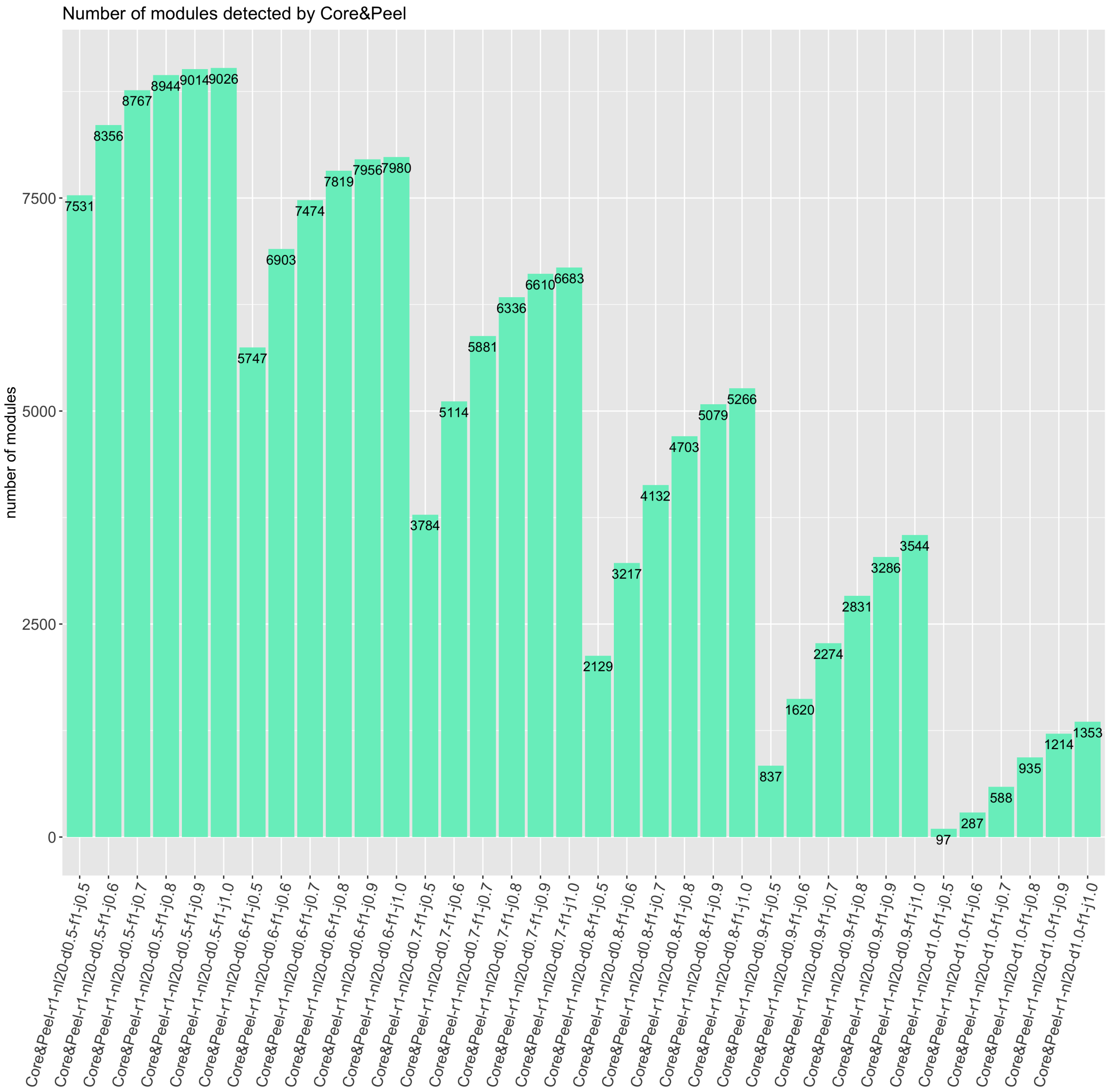

Figure S 1: Number of modules detected by Core&Peel starting from DREAM challenge co-expression network. Different values of density (d) and Jaccard index (j) were tested. In abscissa the tested configurations of parameters for Core&Peel.

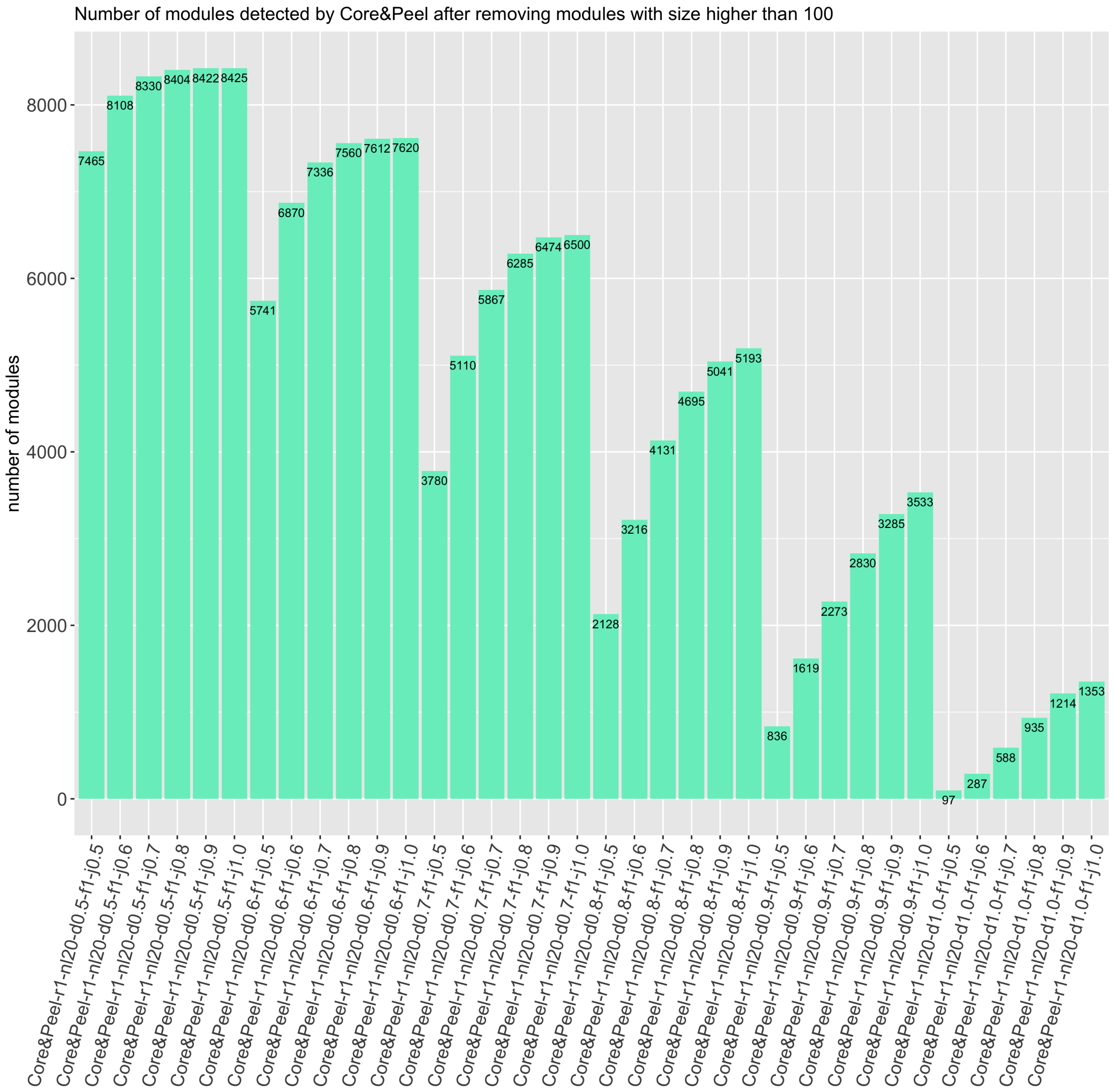

Figure S 2: Number of modules detected by Core&Peel after removing modules with size higher than 100. In abscissa the tested configurations of parameters for Core&Peel

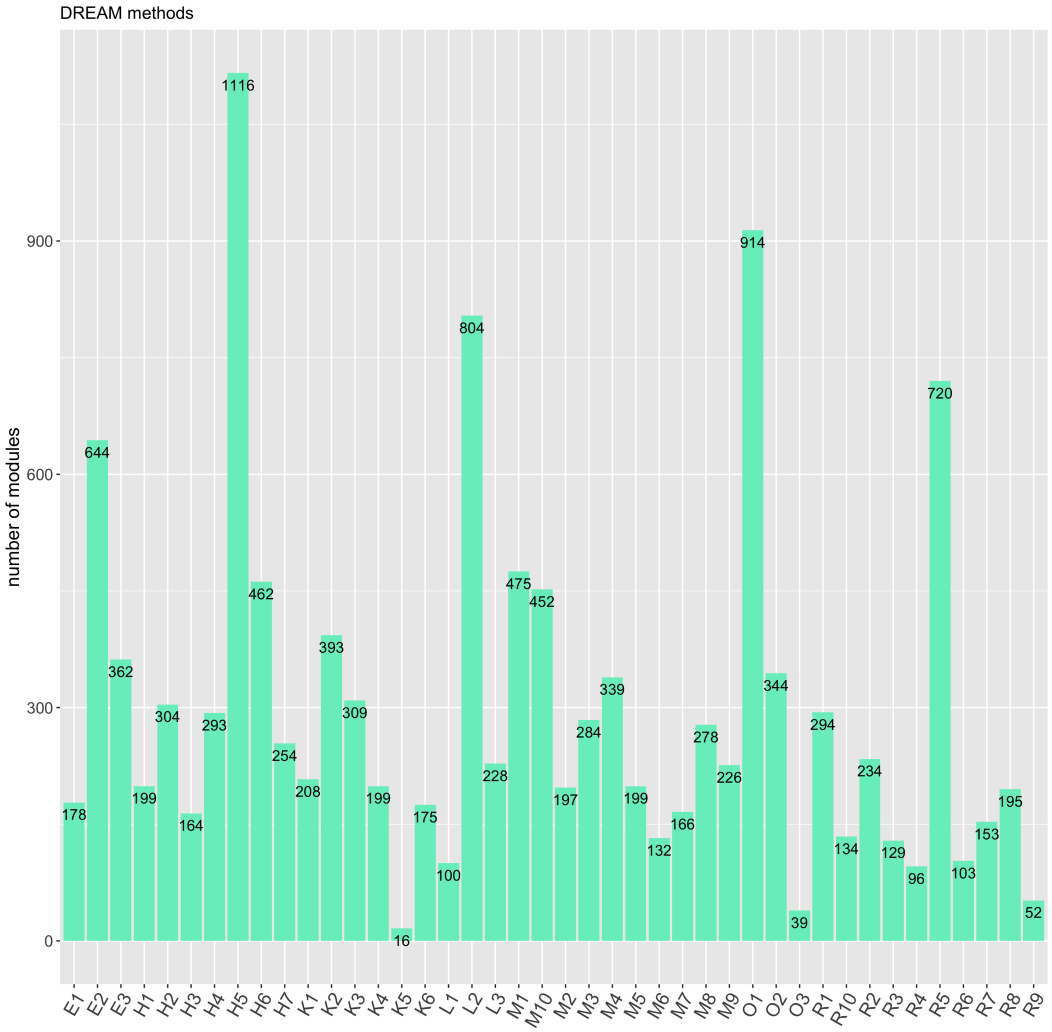

Figure S 3: Number of modules detected by the DREAM methods using the co-expression network.

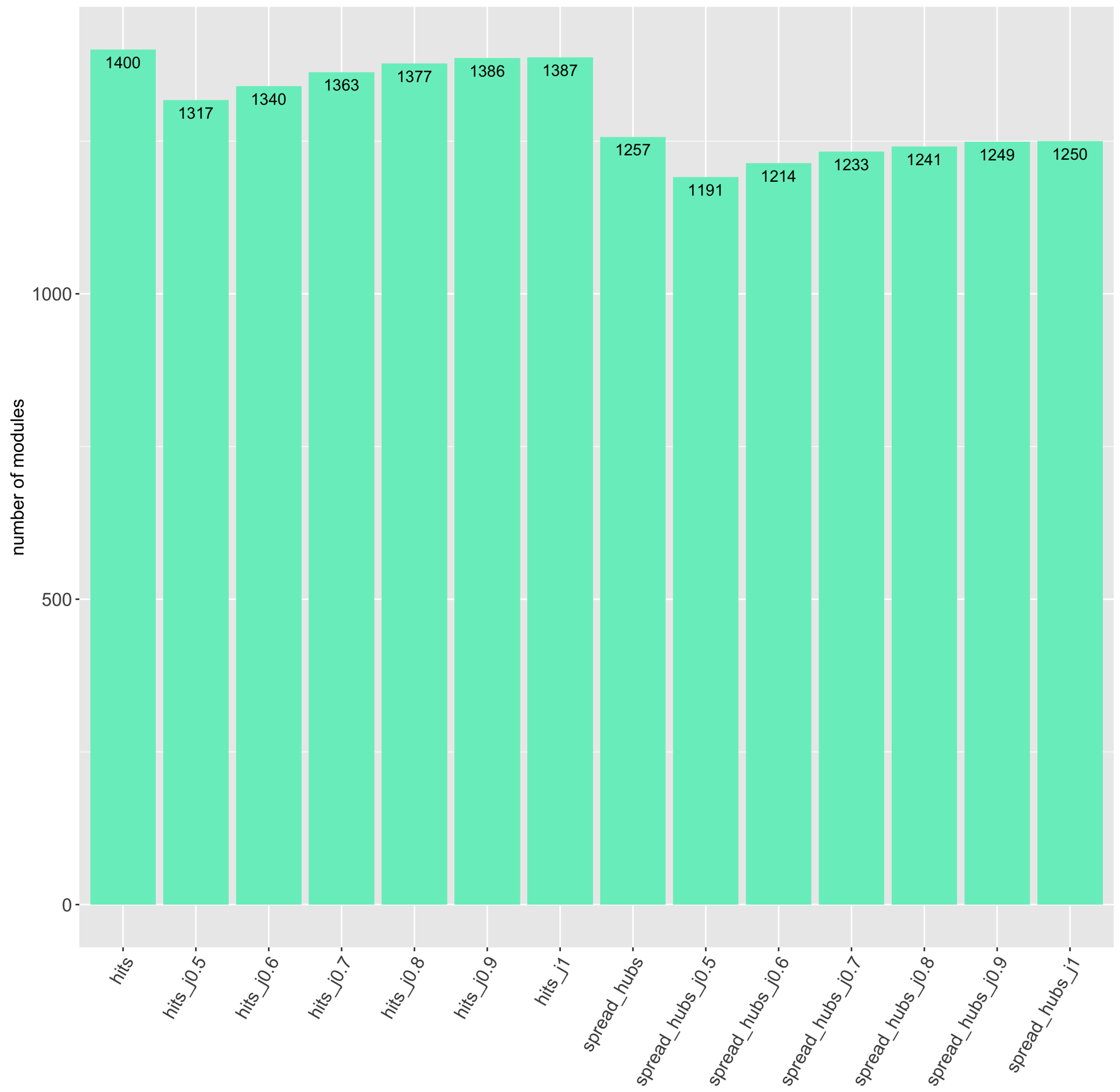

Figure S 4: Number of modules detected by the two options (hits and spread hubs) of the method explained in "A further method: overlapping community detection" section. DREAM challenge co-expression network was used as input. Hits and spread_hubs have not been filtered for Jaccard coefficient.

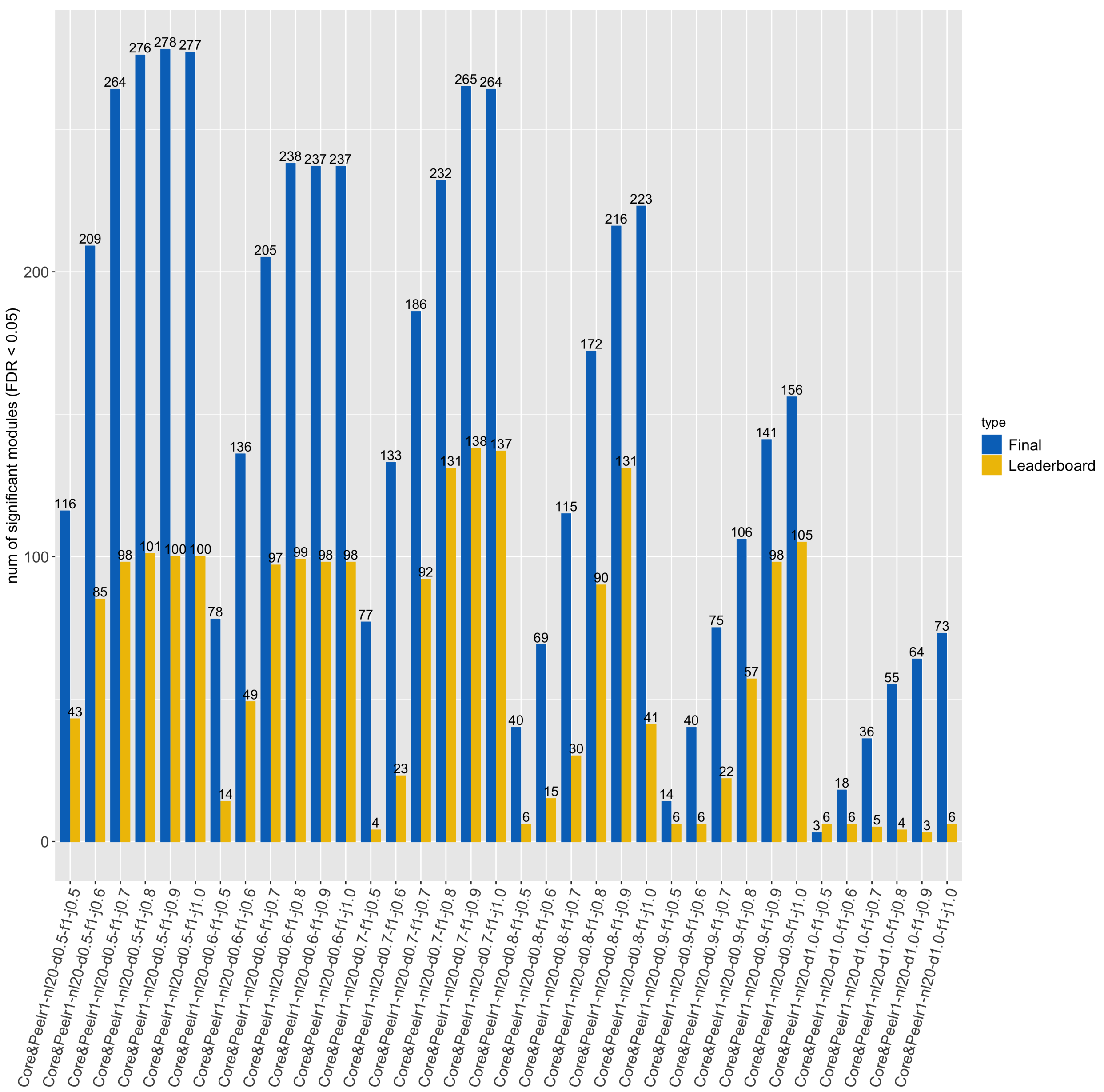

Figure S 5: Number of enriched disease modules detected by Core&Peel using the Leaderboard and Final GWAS datasets, without removing modules with size higher than 100. The number of enriched modules were calculated by the Pascal tool using a 0.05 FDR cutoff.

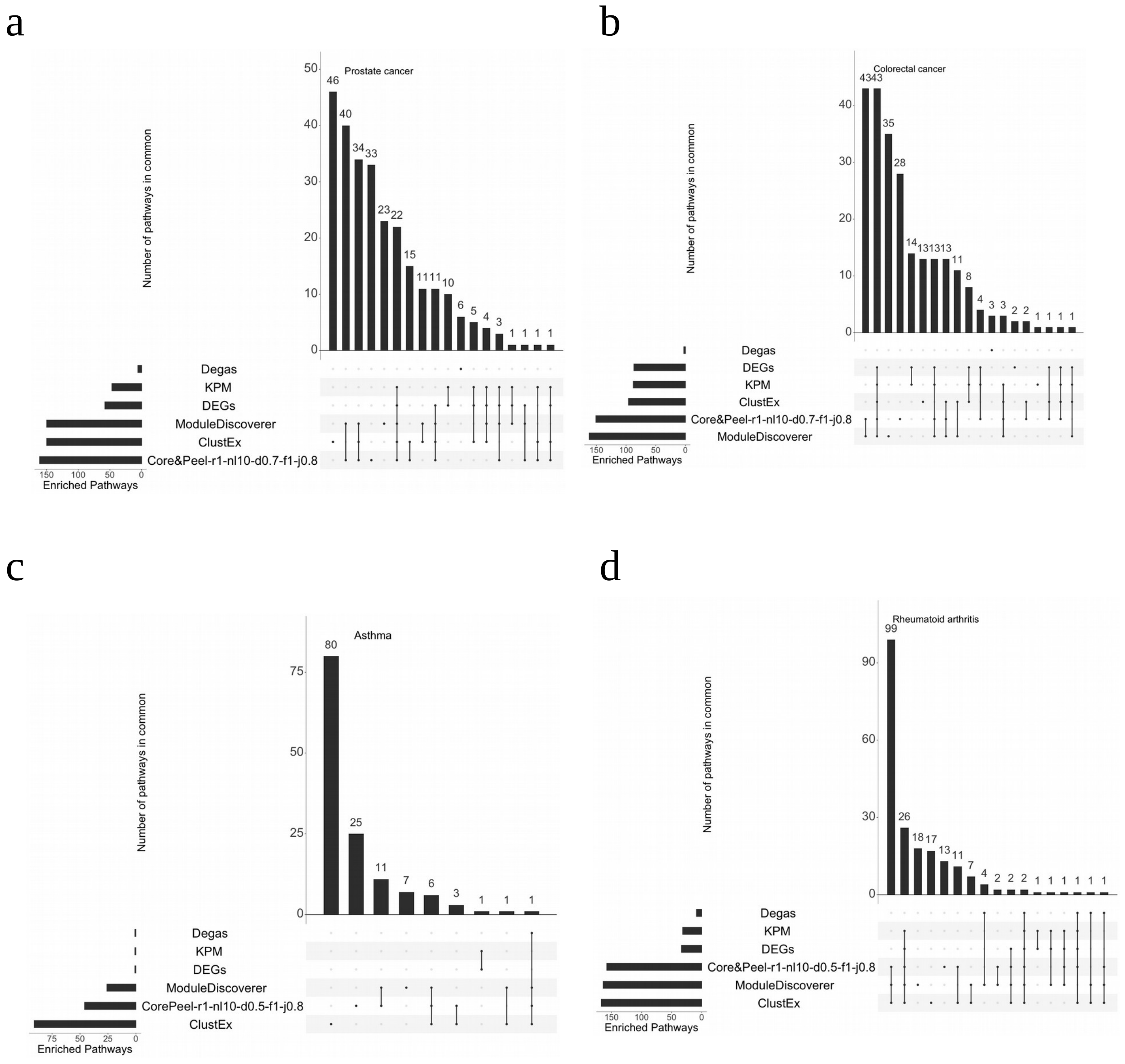

Figure S 6: Overlap of Reactome enriched pathways among all method combinations in prostate cancer (a), colorectal cancer (b), asthma (c) and rheumatoid arthritis (d). The pathways enrichment analysis was performed for each subnetwork detected by Core&Peel and by the four competitive methods (ClustEx, Degas, KPM and ModuleDiscoverer) using Reactome database. The pathway analysis using the differentially expressed genes (DEGs) was conducted as baseline. Only pathways with an adjusted p-vale < 0.05 were selected. The bars on the bottom-left represent the number of enriched pathways in each method. The bars on the main plot represent the number of enriched pathways in common between methods marked with black points on the panel below. The plot was generated by the R package *UpSetR*.

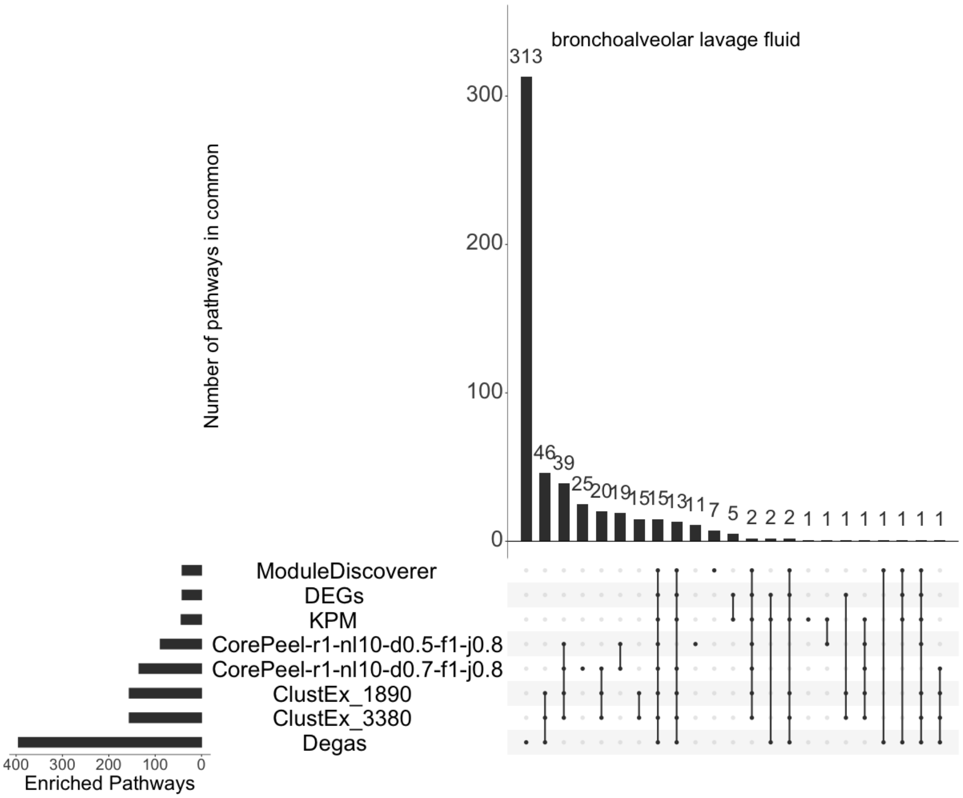

Figure S 7: Overlap of Reactome enriched pathways among all method combinations in COVID-19 BALF case study. The pathways enrichment analysis was performed for each subnetwork detected by Core&Peel and by the four competitive methods (ClustEx, Degas, KPM and ModuleDiscoverer). Both Core&Peel configurations with density equals to 0.5 and 0.7 were tested. Consequently, two versions of ClustEx (ClustEx_1890 and ClustEx_3380) have been generated in order to get modules with size comparable with Core&Peel. The pathway analysis using the differentially expressed genes was conducted as baseline (DEGs). Only pathways with an adjusted p-value < 0.05 were selected. The bars on the bottom-left represent the number of enriched pathways in each method. The bars on the main plot represent the number of enriched pathways in common between methods marked with black points on the panel below. The plot was generated by the R package *UpSetR*.

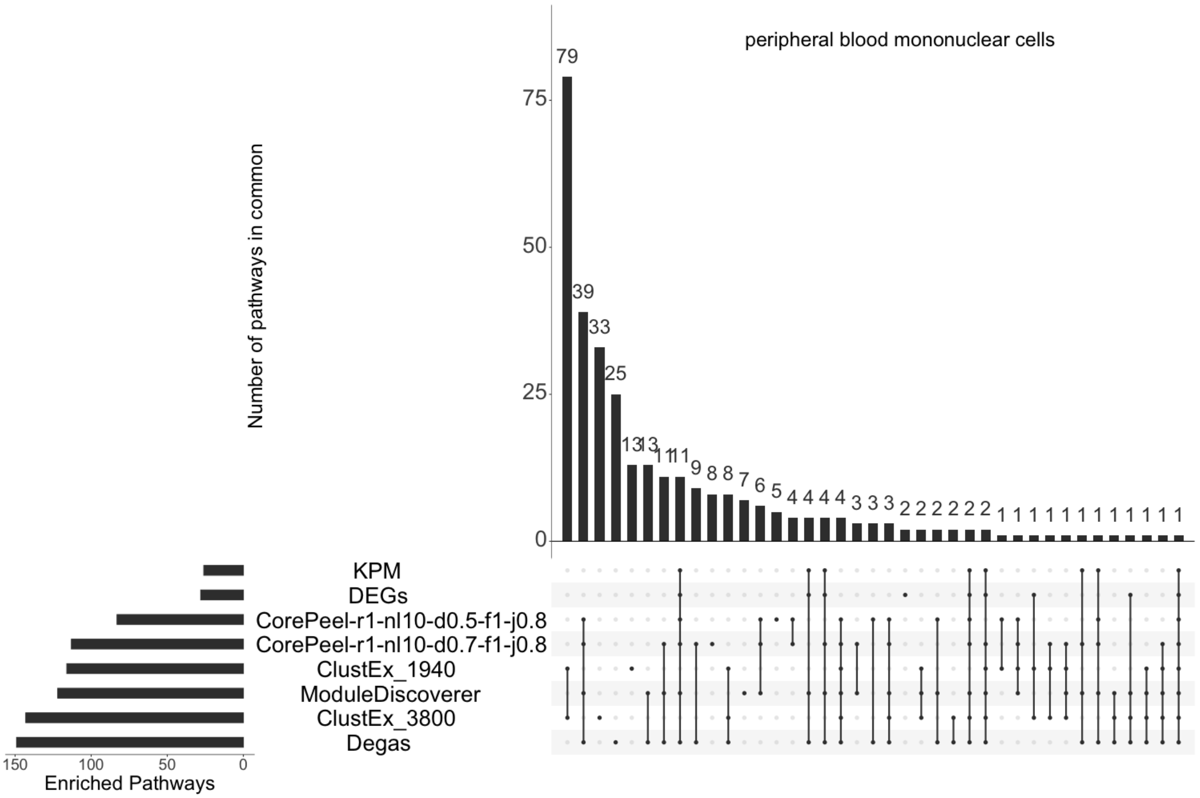

Figure S 8: Overlap of Reactome enriched pathways among all method combinations in COVID-19 PBMC case study. The pathways enrichment analysis was performed for each subnetwork detected by Core&Peel and by the four competitive methods (ClustEx, Degas, KPM and ModuleDiscoverer). Both Core&Peel configurations with density equals to 0.5 and 0.7 were tested. Consequently, two versions of ClustEx (ClustEx_1940 and ClustEx_3800) have been generated in order to get modules with size comparable with Core&Peel. The pathway analysis using the differentially expressed genes (DEGs) was conducted as baseline. Only pathways with an adjusted p-value < 0.05 were selected. The bars on the bottom-left represent the number of enriched pathways in each method. The bars on the main plot represent the number of enriched pathways in common between methods marked with black points on the panel below. The plot was generated by the R package *UpSetR*.

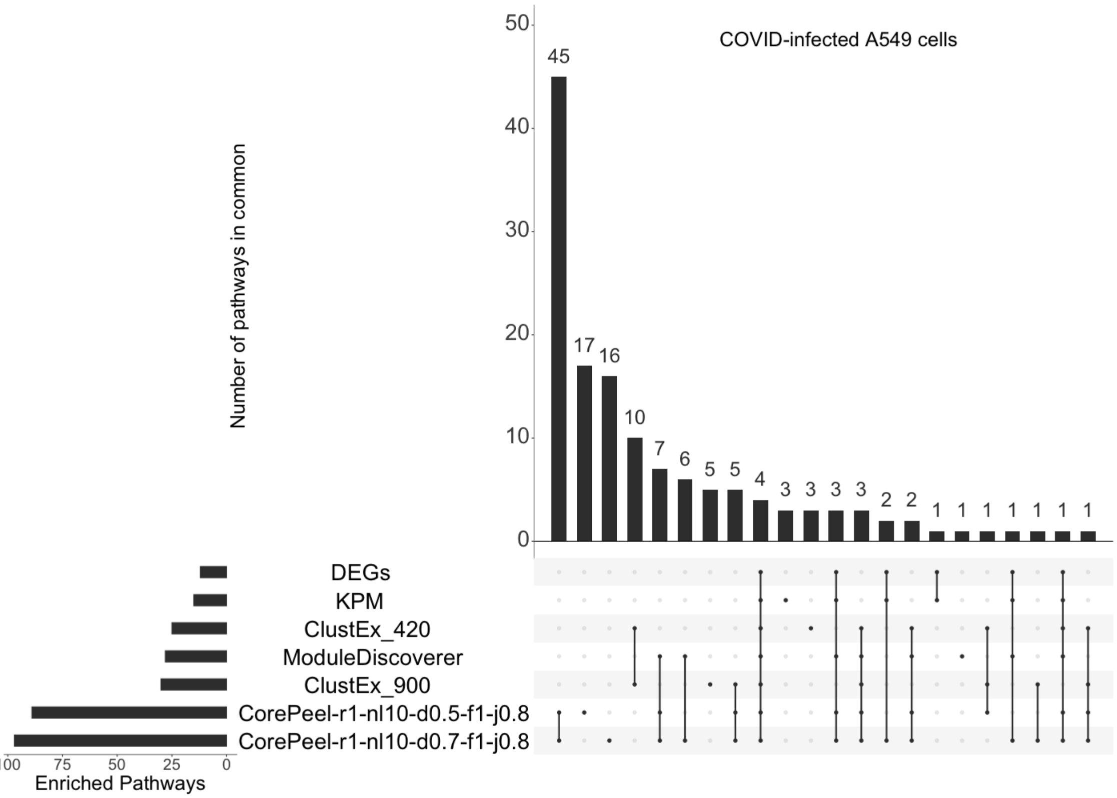

Figure S 9: Overlap of Reactome enriched pathways among all method combinations in COVID-19-infected cells case study. The pathways enrichment analysis was performed for each subnetwork detected by Core&Peel and by the four competitive methods (ClustEx, Degas, KPM and ModuleDiscoverer). Both Core&Peel configurations with density equals to 0.5 and 0.7 were tested. Consequently, two versions of ClustEx (ClustEx_400 and ClustEx_900) have been generated in order to get modules with size comparable with Core&Peel. The pathway analysis using the differentially expressed genes (DEGs) was conducted as baseline. Only pathways with an adjusted p-value < 0.05 were selected. The bars on the bottom-left represent the number of enriched pathways in each method. The bars on the main plot represent the number of enriched pathways in common between methods marked with black points on the panel below. The plot was generated by the R package *UpSetR*.

2 Supplementary Tables

	exp(-2)	exp(-3)	exp(-4)	exp(-5)	exp(-6)	exp(-7)
Core&Peel-r1-nl20-d0.7-f1-j0.8-M6	132	103	64	56	47	43
M6	132	132	107	72	61	54
Core&Peel-r1-nl20-d0.7-f1-j0.8-L2	**804**	673	**449**	**354**	**278**	**230**
L2	800	708	299	155	92	63
Core&Peel-r1-nl20-d0.7-f1-j0.8-K2	393	318	**201**	**168**	**137**	**117**
K2	393	347	165	100	71	51
Core&Peel-r1-nl20-d0.7-f1-j0.8-K5	16	14	9	7	**7**	**7**
K5	16	16	12	7	3	2
Core&Peel-r1-nl20-d0.7-f1-j0.8-M2	197	154	97	84	68	**60**
M2	197	186	139	89	68	57
Core&Peel-r1-nl20-d0.7-f1-j0.8-E1	178	137	84	73	58	**54**
E1	178	172	130	94	64	52
Core&Peel-r1-nl20-d0.7-f1-j0.8-M9	226	180	**108**	**94**	**75**	**66**
M9	226	205	103	66	53	46
Core&Peel-r1-nl20-d0.7-f1-j0.8-R6	**103**	**80**	**51**	**45**	**38**	**37**
R6	102	87	38	31	23	21
Core&Peel-r1-nl20-d0.7-f1-j0.8-M7	166	130	81	70	**55**	**51**
M7	166	160	108	72	49	32
Core&Peel-r1-nl20-d0.7-f1-j0.8-O2	**344**	**282**	**176**	**148**	**119**	**102**
O2	337	281	34	17	10	7
Core&Peel-r1-nl20-d0.7-f1-j0.8-H7	254	**203**	**126**	**107**	**86**	**75**
H7	254	189	1	0	0	0
Core&Peel-r1-nl20-d0.7-f1-j0.8-R3	**129**	100	61	53	43	41
R3	126	114	74	59	52	42
Core&Peel-r1-nl20-d0.7-f1-j0.8-K4	199	157	98	**85**	**69**	**61**
K4	199	188	124	71	56	50
Core&Peel-r1-nl20-d0.7-f1-j0.8-K3	**309**	251	**159**	**136**	**108**	**93**
K3	308	278	150	87	64	49
Core&Peel-r1-nl20-d0.7-f1-j0.8-M5	**199**	157	98	**85**	**69**	**61**
M5	198	185	124	81	55	42
Core&Peel-r1-nl20-d0.7-f1-j0.8-R7	153	119	**74**	**63**	**51**	**47**
R7	153	138	64	44	33	28
Core&Peel-r1-nl20-d0.7-f1-j0.8-R2	234	187	114	**99**	**79**	**69**
R2	234	217	145	82	58	47
Core&Peel-r1-nl20-d0.7-f1-j0.8-H6	**462**	377	**241**	**200**	**161**	**139**
H6	461	391	56	18	9	7
Core&Peel-r1-nl20-d0.7-f1-j0.8-R10	134	**105**	**66**	**57**	**48**	**44**
R10	134	94	1	0	0	0
Core&Peel-r1-nl20-d0.7-f1-j0.8-H5	**1116**	937	**626**	**491**	**377**	**305**
H5	1110	952	201	114	79	59
Core&Peel-r1-nl20-d0.7-f1-j0.8-H4	293	238	151	**130**	**103**	**88**
H4	293	269	180	91	67	38
Core&Peel-r1-nl20-d0.7-f1-j0.8-R4	96	75	47	41	35	34
R4	96	88	58	44	37	34
Core&Peel-r1-nl20-d0.7-f1-j0.8-M10	**452**	368	**235**	**195**	**156**	**134**
M10	451	414	154	71	56	34
Core&Peel-r1-nl20-d0.7-f1-j0.8-M4	339	279	176	**148**	**119**	**102**

M4	339	310	187	114	80	63
Core&Peel-r1-nl20-d0.7-f1-j0.8-H1	199	157	98	85	69	61
H1	199	189	149	107	74	57
Core&Peel-r1-nl20-d0.7-f1-j0.8-R5	720	604	**405**	**323**	**252**	**213**
R5	720	652	227	130	77	63
Core&Peel-r1-nl20-d0.7-f1-j0.8-O1	914	766	**514**	**407**	**316**	**257**
O1	914	799	116	62	41	34
Core&Peel-r1-nl20-d0.7-f1-j0.8-O3	39	**35**	**20**	**18**	**16**	**16**
O3	39	27	0	0	0	0
Core&Peel-r1-nl20-d0.7-f1-j0.8-H3	164	128	79	68	**54**	**50**
H3	164	159	115	77	51	39
Core&Peel-r1-nl20-d0.7-f1-j0.8-M3	284	230	144	**123**	**98**	**84**
M3	284	265	180	100	75	62
Core&Peel-r1-nl20-d0.7-f1-j0.8-L1	**100**	77	48	42	36	35
L1	99	90	62	54	49	40
Core&Peel-r1-nl20-d0.7-f1-j0.8-K1	208	165	101	88	70	**63**
K1	208	201	154	90	70	56
Core&Peel-r1-nl20-d0.7-f1-j0.8-R1	294	238	151	**130**	**103**	**88**
R1	294	264	175	111	84	67
Core&Peel-r1-nl20-d0.7-f1-j0.8-M1	475	390	**248**	**207**	**167**	**145**
M1	474	416	167	97	78	61
Core&Peel-r1-nl20-d0.7-f1-j0.8-H2	**304**	246	156	**134**	**106**	**91**
H2	303	280	182	127	92	67
Core&Peel-r1-nl20-d0.7-f1-j0.8-E3	362	296	**188**	**157**	**127**	**108**
E3	362	315	109	52	35	22
Core&Peel-r1-nl20-d0.7-f1-j0.8-E2	**644**	537	**352**	**286**	**226**	**194**
E2	643	580	232	126	77	60
Core&Peel-r1-nl20-d0.7-f1-j0.8-L3	228	182	111	96	**77**	**68**
L3	228	216	126	94	71	57
Core&Peel-r1-nl20-d0.7-f1-j0.8-K6	**175**	**134**	**82**	**71**	**56**	**52**
K6	173	130	39	21	12	9
Core&Peel-r1-nl20-d0.7-f1-j0.8-R8	**195**	152	**96**	**83**	**66**	**60**
R8	194	166	83	56	45	38
Core&Peel-r1-nl20-d0.7-f1-j0.8-R9	52	44	25	**23**	**20**	**19**
R9	52	46	25	18	13	10
Core&Peel-r1-nl20-d0.7-f1-j0.8-M8	278	223	140	**119**	**96**	**82**
M8	278	245	164	104	70	54
mean Core&Peel	**299.1**	243.5	**156**	**129.7**	**103.4**	**89.2**
mean DREAM	298.4	265.7	118.1	71.5	51.3	40

Table S 1: Summary of the GO enrichment analysis results. The values shown represent the number of predicted modules with GO enrichment fdr below different thresholds. Core&Peel-r1-nl20-d0.7-f1-j0.8 was compared with DREAM methods after applying the CRank algorithm to Core&Peel in order to get the same number of modules. When Core&Peel detects more enriched modules than DREAM methods the corresponding number is in bold. The last row represents the mean of the enriched modules for Core&Peel and DREAM methods for each threshold.

	exp(-2)	exp(-3)	exp(-4)	exp(-5)	exp(-6)	exp(-7)
Core&Peel-r1-nl20-d0.7-f1-j0.8	**6285**	**5332**	**3343**	**2169**	**1445**	**1063**
hits jaccard0.8	1377	953	346	189	111	88
hits	1400	975	362	206	117	95
spread hubs jaccard0.8	1241	854	329	169	108	87
spread hubs	1257	870	345	183	114	91

Table S 2: Summary of the GO enrichment analysis results comparing Core&Peel-r1-nl20-d0.7-f1-j0.8 with the method developed by Raman group which detects overlapping communities. The values shown represent the number of predicted modules with GO enrichment fdr below different thresholds. Hits jaccard0.8 and spread hubs jaccard0.8 are filtered in order to have the maximum Jaccard separation equal to 0.8; hits and spread hubs are the same methods but without any filtering. The numbers in bold highlight the best method in detecting more significant GO terms.

	exp(-2)	exp(-3)	exp(-4)	exp(-5)	exp(-6)	exp(-7)
Core&Peel-r1-nl20-d0.7-f1-j0.8	1	**0.848**	**0.532**	**0.345**	**0.230**	**0.169**
hits jaccard0.8	1	0.692	0.251	0.137	0.08	0.064
hits	1	0.696	0.258	0.147	0.083	0.068
spread hubs jaccard0.8	1	0.688	0.265	0.136	0.087	0.07
spread hubs	1	0.692	0.274	0.145	0.09	0.072

Table S 3: Fraction of modules with GO enrichment fdr below different thresholds. We are comparing Core&Peel-r1-nl20-d0.7-f1-j0.8 with the method developed by Raman group (hits and spread hubs options). Hits jaccard0.8 and spread hubs jaccard0.8 are filtered in order to have the maximum Jaccard separation equal to 0.8; hits and spread hubs are the same methods but without any filtering. The numbers in bold highlight the best method in detecting more significant GO terms.

	case	control	DEGs
Prostate cancer	478	52	1301
Asthma	12	15	100
Rheumatoid	18	15	21
Colorectal cancer	451	41	2995
BALF	2	3	630
PBMC	3	3	538
COVID19-cells	not specified	not specified	38

Table S 4: Summary of the transcriptomic data for each case study: prostate cancer, asthma, rheumatoid arthritis, colorectal cancer and the three COVID-19 datasets (BALF, PBMC and COVID19-cells). In each study, case patients are compared with control samples. Case: number of samples with disease; control: number of healthy samples; DEGs: number of differentially expressed genes included in the co-expression network. In COVID19-cells case study, the number of cases and controls were not reported.

	BALF	PBMC	COVID-cells
Core&Peel-r1-nl10-d0.5-f1-j0.8	3380	3809	904
Core&Peel-r1-nl10-d0.7-f1-j0.8	1895	1943	423
MD	1183	1329	273
Degas	4112	122	-
ClustEx	3380-1890	3800-1940	900-420
KPM	605	520	38

Table S 5: Number of genes in each active subnetwork in the three COVID-19 cases. Both Core&Peel configurations have been tested. Two versions of ClustEx have been generated in order to get modules with size comparable with Core&Peel. It was not possible to run Degas in COVID-cells case since the expression matrix was not available.

**Pathway**	**Core&Peel d0.5-j0.8**	**Core&Peel d0.7-j0.8**	**MD**	**ClustEx 3380**	**ClustEx 1890**	**KPM**	**Degas**	**DEGs**
Staphylococcus aureus infection	1.42	NA	NA	NA	NA	NA	NA	NA
Inflammatory mediator regulation of TRP channels	1.31	NA	NA	NA	NA	NA	NA	NA
Cytokines and Inflammatory Response WP530	NA	NA	1.35	1.68	2.0	NA	NA	NA
Ebola Virus Pathway on Host WP4217	NA	NA	1.90	6.02	5.0	NA	6.48	NA
Human papillomavirus infection	NA	NA	NA	2.38	4.2	NA	NA	NA
Vibrio cholerae infection	NA	NA	NA	2.38	2.63	NA	1.88	NA
Epstein-Barr virus infection	NA	NA	NA	2.12	1.84	NA	3.88	NA
Kaposi sarcoma-associated herpesvirus infection	NA	NA	NA	1.34	3.28	NA	2.44	NA
Human cytomegalovirus infection	NA	NA	NA	1.33	3.10	NA	1.96	NA
Epithelial cell signaling in Helicobacter pylori infection	NA	NA	NA	NA	2.53	NA	2.87	NA
Yersinia infection	NA	NA	NA	NA	2.39	NA	2.67	NA
Human immunodeficiency virus 1 infection	NA	NA	NA	NA	1.95	NA	1.47	NA
Salmonella infection	NA	NA	NA	NA	1.81	NA	6.57	NA
T-Cell antigen Receptor (TCR) pathwayduring Staphylococcus aureus infection WP3863	NA	NA	NA	3.08	2.89	NA	4.19	NA
Pathogenic Escherichia coli infection	NA	NA	NA	NA	NA	NA	1.94	NA
IL-10 Anti-inflammatory Signaling Pathway WP4495	NA	NA	NA	NA	NA	NA	1.33	NA

Table S 6: Most interesting enriched pathways in BALF-COVID19 case. The negative adjusted p-values in log10-scale are reported. If one pathway is not significant in a method, NA is reported. Both Core&Peel configurations (Core&Peel-r1-nl10-d0.5-f1-j0.8 and Core&Peel-r1-nl10-d0.7-f1-j0.8) have been tested. Consequently two versions of ClustEx have been generated in order to get them comparable with the two Core&Peel configurations according to the different module sizes. MD: ModuleDiscoverer; KPM: KeyPathwayMiner; DEGs: Differentially Expressed Genes.

**Pathways**	**Core&Peel d0.5-j0.8**	**Core&Peel d0.7-j0.8**	**MD**	**KPM**	**ClustEx 3800**	**ClustEx 1940**	**Degas**	**DEGs**
Staphylococcus aureus infection	NA	2.60	NA	NA	NA	1.74	NA	NA
Human immunodeficiency virus infectious disease	5.72	5.82	3.87	1.49	NA	NA	NA	NA
Inflammatory Response Pathway WP453	3.89	5.16	NA	NA	1.66	1.45	NA	NA
T-Cell antigen Receptor (TCR) pathway during Staphylococcus aureus infection WP3863	2.53	NA	NA	NA	2.75	1.48	NA	NA
Pathogenic Escherichia coli infection WP2272	2.18	NA	NA	NA	NA	NA	NA	NA
Ebola Virus Pathway on Host WP4217	1.49	NA	NA	NA	5.77	4.64	NA	NA
Epstein-Barr virus infection	NA	NA	NA	NA	3.19	2.53	NA	NA
Vibrio cholerae infection	NA	NA	NA	NA	NA	2.02	NA	NA
Epithelial cell signaling in Helicobacter pylori infection	NA	NA	NA	NA	NA	1.37	NA	NA
Human papillomavirus infection	NA	NA	NA	NA	2.29	NA	NA	NA
Cytokines and Inflammatory Response WP530	NA	NA	NA	NA	3.80	1.77	NA	NA
IL-10 Anti-inflammatory Signaling Pathway WP4495	NA	NA	NA	NA	1.38	NA	NA	NA

Table S 7: Most interesting enriched pathways in PBMC-COVID19 case. The negative adjusted p-values in log10-scale are reported. If one pathway is not significant, NA is reported. Both Core&Peel configurations (Core&Peel-r1-nl10-d0.5-f1-j0.8 and Core&Peel-r1-nl10-d0.7-f1-j0.8) have been tested. Consequently two versions of ClustEx have been generated in order to get them comparable with the two Core&Peel configurations according to the different module sizes. MD: ModuleDiscoverer; KPM: KeyPathwayMiner; DEGs: Differentially Expressed Genes.

Pathway	Core&Peel d0.5-j0.8	Core&Peel d0.7-j0.8	MD	ClustEx 900	ClustEx 420	KPM	DEGs
positive regulation of cytokine production involved in inflammatory response	1.57	2.96	NA	NA	NA	NA	NA
regulation of cytokine production involved in inflammatory response	2.14	2.82	NA	NA	NA	NA	NA
cytokine production involved in inflammatory response	1.90	2.62	NA	NA	NA	NA	NA
Inflammatory mediator regulation of TRP channels	2.14	1.94	NA	NA	NA	NA	NA
IL-10 Anti-inflammatory Signaling Pathway WP4495	2.87	2.87	NA	NA	NA	NA	NA
Cytokines and Inflammatory Response WP530	1.49	NA	NA	2.8	1.62	NA	NA
Recurrent Staphylococcus aureus infections	1.42	2.72	NA	NA	NA	NA	NA
Epstein-Barr virus infection	10.16	13.69	6.18	6.70	7.84	7.33	7.33
Human immunodeficiency virus 1 infection	7.19	7.43	2.75	1.78	3.88	NA	NA
Herpes simplex virus 1 infection	4.60	7.28	4.31	3.32	4.05	5.73	5.73
Staphylococcus aureus infection	4.54	1.94	4.99	2.75	NA	NA	NA
Human cytomegalovirus infection	4.47	3.0	2.95	NA	1.38	NA	NA
Salmonella infection	2.23	3.29	NA	NA	NA	NA	NA
Yersinia infection	2.21	NA	1.82	NA	NA	NA	NA
Human papillomavirus infection	1.90	2.29	2.82	NA	1.37	2.81	2.81
Pathogenic Escherichia coli infection	1.75	2.77	NA	NA	1.74	NA	NA
T-Cell antigen Receptor (TCR) pathway during Staphylococcus aureus infection WP3863	4.24	3.90	2.35	NA	NA	NA	NA
Ebola Virus Pathway on Host WP4217	8.36	7.07	4.99	4.85	3.05	1.91	1.59

Table S 8: Most interesting enriched pathways in COVID19-cells case. The negative adjusted p-values in log10-scale are reported. If one pathway is not significant, NA is reported. Both Core&Peel configurations (Core&Peel-r1-nl10-d0.5-f1-j0.8 and Core&Peel-r1-nl10-d0.7-f1-j0.8) have been tested. Consequently two versions of ClustEx have been generated in order to get them comparable with the two Core&Peel configurations according to the different module sizes. MD: ModuleDiscoverer; KPM: KeyPathwayMiner; DEGs: Differentially Expressed Genes.

3 Supplementary Notes

3.1 Algorithms for active subnetwork identification

We tested four existing active subnetwork identification algorithms. We selected the methods that take gene expression matrix and/or list of differentially expressed genes (DEGs) as their inputs [1], a part from the network (generally it is a protein-protein interaction network). In this case, we used the co-expression network by the DREAM challenge as input. The algorithms we used in the comparison are summarized as follows.

ModuleDiscoverer [2]: Starting from a network, the algorithm first approximates the underlying community structure by iterative enumeration of cliques from random seed nodes (one or more) in the network. Secondly, all the cliques are tested for their enrichment with the DEGs obtained from the gene expression data. Finally, significantly enriched cliques are assembled in a unique regulatory module taking each gene once.
We used the single-seed approach, which identifies cliques using only one seed node and the p-value cutoff was 0.01 (both default parameters). The p-value for each clique is calculated using a permutation-based test. To calculate this p-value we used the parameter of 20000 gene-sets.

KeyPathwayMiner (KPM) [3]: it detects highly connected subnetworks in which most genes show similar patterns of expression. Specifically, given network and gene expression data, KeyPathwayMiner identifies those maximal subgraphs where all but k nodes of the subnetwork are expressed similarly in all but l cases in the gene expression data. We provided an indicator flag to mark differentially expressed genes (0/1). We set k= 2 and l = 0 and INES strategy with GREEDY algorithm was performed. More modules were detected but most of them differs from one or two genes, so the best-scoring module was selected for further analysis.

DEGAS [4]: it identifies connected gene subnetworks significantly enriched for genes that are dysregulated in samples with disease. It takes the network and the gene expression matrix as inputs and each sample is labeled as case or control. We used default parameters a part from l (the number of outlier cases) which was set as 20% of the case number (as suggested by the authors). The algorithm identified one regulatory module for case study, which were used for further analysis. Only in BALF and PBMC cases, it detected more than one module so we merged them together. Beside it was not possible to run Degas in COVID-cells case since the expression matrix was not available.

ClustEx [5]: the algorithm takes DEGs and network as input. The DEGs are clustered and partitioned into different groups by average linkage hierarchical clustering according to their distances in the network. In the extending step, the final modules are formed by adding intermediate genes on the k-shortest paths between the DEGs. The size of the largest module has to be chosen. The output consists of some small modules (about 2 or 3 genes for each) and one big module. We took the biggest module for the downstream analyses. The size of the largest module was chosen in order to be comparable with the module size of Core&Peel.

3.2 Core&Peel: detection of active sub-network when nl = 20

Here we show the Core&Peel performance in detecting active subnetwork when nl = 20. We chose the Core&Peel-r1-nl20-d0.8-f1-j0.8 for the prostate and colorectal cancer cases, instead Core&Peel-r1-nl20-d0.5-f1-j0.8 in asthma and rheumatoid arthritis case. Core&Peel detected 743, 120, 33 and 837 modules in the prostate cancer, asthma, rheumatoid arthritis and colorectal cancer datasets, respectively. Since the significant modules were merged in one regulatory module, we checked if these modules were still significantly enriched with the DEGs; all the four modules got a p-value $< 10^{-12}$.

3.2.1 Comparison with other active sub-network detection methods

We compared the Core&Peel modules with those detected by the four existing active subnetwork identification methods, namely ModuleDiscoverer (MD), KeyPathwayMiner (KPM), ClustEx and Degas. We also tested MD using the hypergeometric exact test (MD_hyperg) instead of the permutation-based test.
The number of genes in each module is reported in Table 9. Also in this case, regulatory modules of asthma and rheumatoid arthritis include a smaller number of genes than the prostate and colorectal cancer modules. Besides when we apply the hypergeometric test to MD, smaller modules have been identified than classic MD.

	Prostate	Asthma	Colorectal	Rheumatoid arthritis
Core&Peel	1679	1170	2220	416
MD	2708	192	2606	403
MD_hyperg	1435	173	1647	160
KPM	1266	74	2965	20
ClustEx	1700	1200	2300	350
Degas	122	37	44	85

Table S 9: Number of genes in each active subnetwork detected by all the methods for prostate and colorectal cancer, asthma and rheumatoid arthritis. Core&Peel-r1-nl20-d0.8-f1-j0.8 configuration was used for prostate and colorectal cancer, instead Core&Peel-r1-nl20-d0.5-f1-j0.8 for asthma and rheumatoid. MD_hyperg is ModuleDiscoverer method using the hypergeometric test instead of the permutation-based test

Next, we compared the enriched pathways (Reactome-based) and plotted the overlap among all the method combinations (Figure 10). In this comparison we took into account the MD_hyperg. Globally, MD_hyperg, ClustEx and Core&Peel detected higher number of pathways than Degas, KPM and DEGs. In both cancer cases, Core&Peel and MD_hyperg share almost half of their pathways (around 60) and a large number of pathways was identified only by ClustEx (Figures 10A, 10B). In rheumatoid arthritis case, more than half of the pathways were shared by Core&Peel, ClustEx and MD_hyperg (Figure 10D) and there is a small overlap for the other combinations. Besides, a smaller number of enriched pathways were detected in asthma than the other three case studies and we can also notice there is a few overlap. In fact almost the entire set of enriched pathways of ClustEx is not in common with any other methods. Similarly, more than half of the pathways identified by Core&Peel, were not detected by the others (Figure 10C).

3.2.2 Detection of disease-associated pathways

We calculated the number of enriched pathways with at least two genes associated to the specific disease (annotated on DisGeNET database). Also in this case we took into account MD_hyperg. The results are showed in Figure 11. Generally Core&Peel is able to identify a substantial number of pathways. In particular, it is able to detect the major number of pathways with disease-associated genes in colorectal cancer and asthma. Generally applying the hypergeometric test reduces the number of pathways with disease-associated-genes, more evident in the rheumatoid arthritis case (Figure 11B).

Finally, we conducted further enrichment analyses using different databases in order to investigate the enrichment with specific-disease pathways. The results are showed in Figure 12. The results for carcinoma and asthma pathways are similar to those obtained for nl = 10 (compare Figure 12 A and B with the corresponding Figure in the main text). The Core&Peel modules are the unique ones to be significantly enriched for colon and prostate cancer specific pathways such as neoplasm of the colon (Figure 12C) and abnormal prostate morphology, prostate cancer and prostate neoplasm (Figure 12D). This highlights how Core&Peel (with nl = 20) was able to find more cancer type-specific modules than the others algorithms. Again no significantly enriched pathways were found for rheumatoid arthritis case.

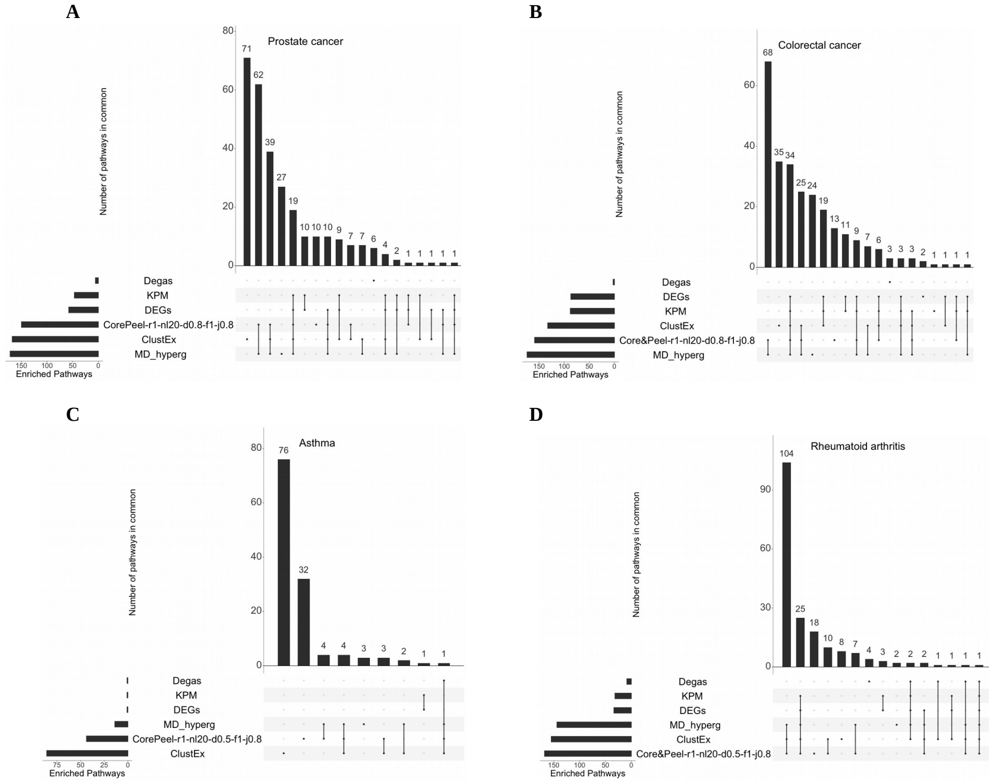

Figure S 10: Overlap of Reactome enriched pathways among all method combinations in prostate cancer (A), colorectal cancer (B), asthma (C) and rheumatoid arthritis (D). The pathway enrichment analysis was performed for each active subnetwork detected by Core&Peel (nl = 20) and by the four competitive methods (ClustEx, Degas, KPM and ModuleDiscoverer). MD_hyperg is ModuleDiscoverer method using the hypergeometric test instead of the permutation-based test. The bars on the bottom-left represent the number of enriched pathways detected by the methods. The bars on the main plot represent the number of enriched pathways in common between methods marked with black points on the panel below. The plot was generated by the R package *UpSetR*.

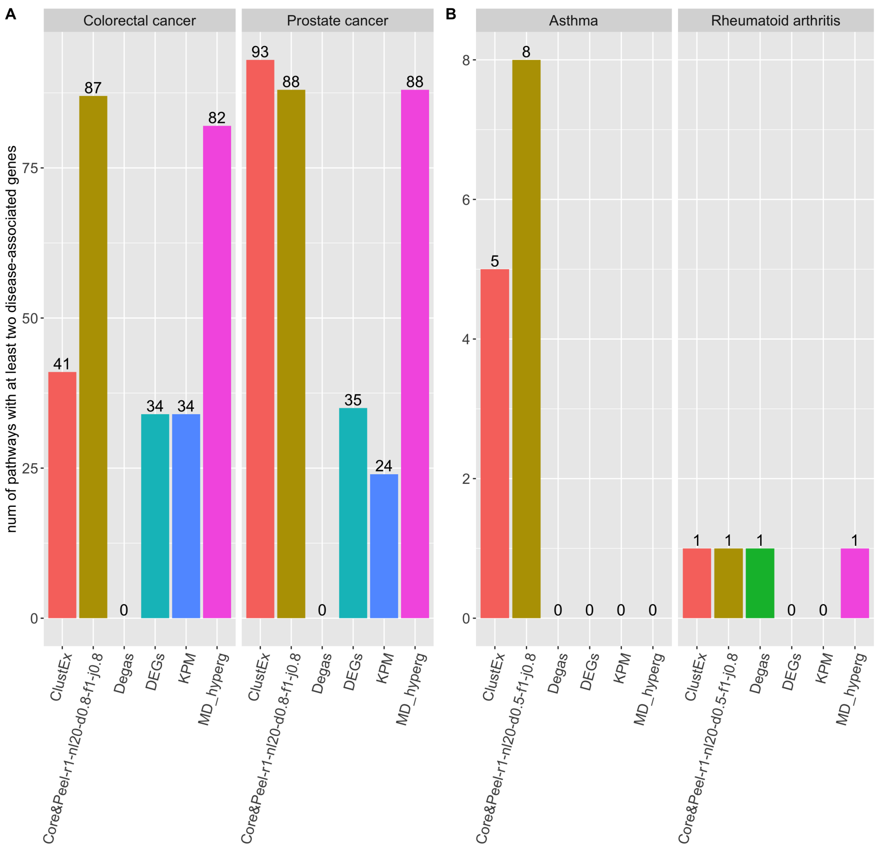

Figure S 11: Number of enriched pathways with at least two disease-associated genes. The pathway enrichment analysis was performed for each subnetwork detected by Core&Peel and by the four competitive methods (ClustEx, Degas, KPM and MD_hyperg) using the Reactome database. The pathway analysis using the differentially expressed genes was conducted as baseline. Pathways with an adjusted p-value < 0.05 were selected and the number of disease-gene association was exctracted by the DisGeNET database. (A) Prostate and Colorectal cancer cases; Core&Peel-r1-nl20-d0.8-f1-j0.8 was used. (B) Asthma and rheumatoid arthritis cases where Core&Peel-r1-nl20-d0.5-f1-j0.8 configuration was used.

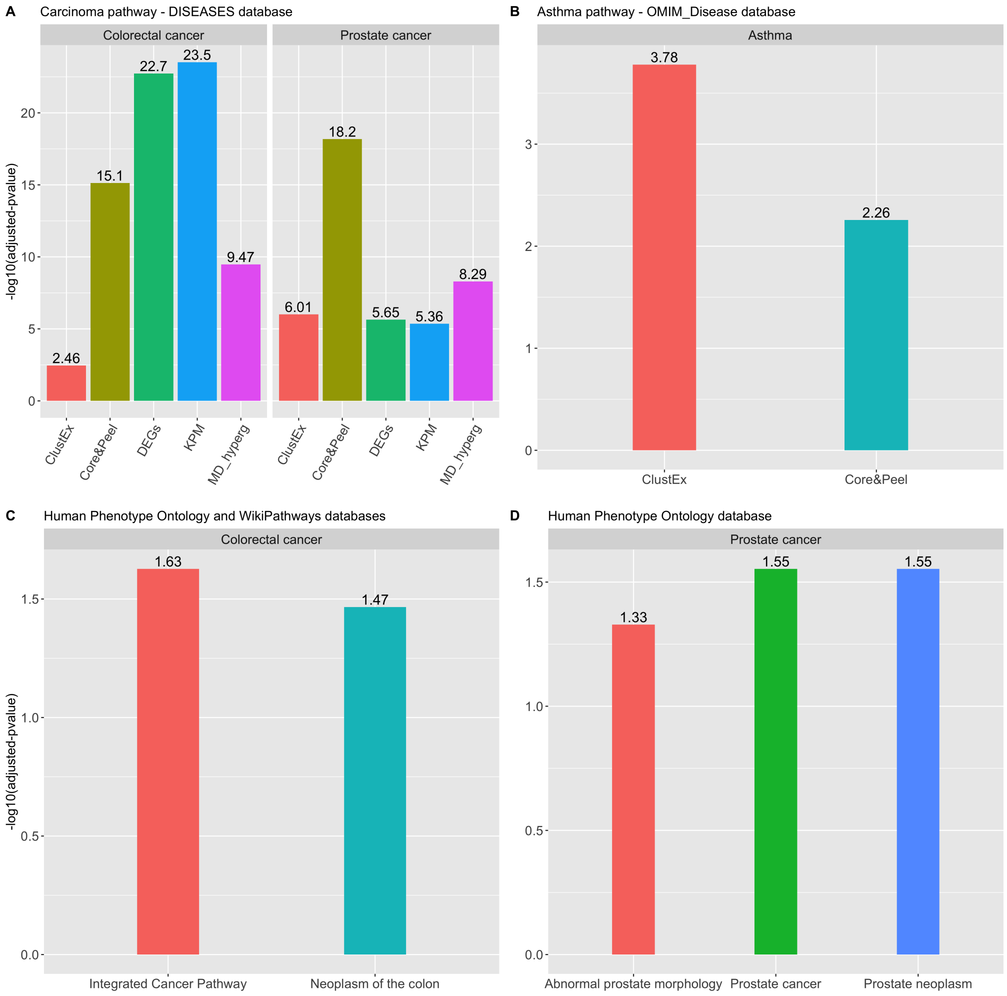

Figure S 12: Results of *gProfileR* and *enrichR* analyses. The bars represent the adjusted p-value in log10-scale. Only the methods which reached the significant p-value (< 0.05) are showed. (A) The adjusted p-values of Carcinoma pathway annotated in DISEASES database in colorectal and prostate cancer. (B) Adjusted p-values of asthma pathway annotated in OMIM_Disease database. Only Core&Peel and ClustEx active subnetworks are enriched for this pathway. (C) Adjusted p-values of Integrated Cancer Pathway and Neoplasm of colon pathways annotated in WikiPathways and Human Phenotype Ontology databases, respectively. Only Core&Peel subnetwork is enriched for these pathways in colorectal cancer. (D) Adjusted p-values of Abnormal prostate morphology, Prostate cancer and neoplasm pathways annotated in Human Phenotype Ontology database. Only Core&Peel module is enriched for these pathways in prostate cancer.

3.3 ClueGO analysis

In order to analyze the pathways detected only by Core&Peel in the active subnetwork identification problem, we used the ClueGO [6], a Cytoscape plug-in which creates a functionally organized pathway term network. This enables us to study the most representative pathways identified by only Core&Peel. For each case study, we selected the Reactome enriched pathways detected only by Core&Peel (no overlap with any other methods) as ClueGO input. Basically a Reactome term-term similarity is calculated (for each pair of terms) using the kappa score, which takes into account the shared genes between the terms. Finally, the created network represents the terms as nodes which are linked based on a predefined kappa score level (default: 0.1). All the terms are iteratively compared and functional groups are defined. Each functional group is represented by its most significant term and the size of the nodes reflects the enrichment significance of the terms. The following networks show the Reactome-pathways identified only by Core&Peel across the four case studies and the tables summarize the representative terms of each group.

3.3.1 Prostate cancer

The majority of the pathways detected in prostate cancer are related to the regulation of the cell cycle and DNA repair, which are the main deregulated processes in cancer. In particular, one of the pathways that plays a role in the cell cycle is associated to the kinetochore, a protein that links the chromosome to microtubule and it is essential for proper chromosome segregation during mitosis. A study [7] showed that the expression of one kinetochore–associated protein was remarkably upregulated in prostate cancer. Knockdown of this protein repressed the ability of cell proliferation, migration, and invasion of prostate cancer cells. Another pathway with a role in the mitosis control is called *AURKA Activation by TPX2*. The protein TPX2 activates Aurora A kinase (AURKA) which contributes to the regulation of cell cycle progression. It has been demonstrated [8] that overexpression of TPX2 improved proliferative, invasive and migratory abilities, and repressed apoptosis of the prostate cancer cells. Pathways involved in the DNA repair processes are for example *Resolution of D-loop Structures through Synthesis-Dependent Strand Annealing (SDSA)* and *HDR through Homologous Recombination (HRR)*. The SDSA and the Homology directed repair (HDR) are mechanisms in cells to repair double-strand DNA lesions. The SDSA is promoted by the DNA helicase and inactivating mutations in DNA helicase genes are frequently associated with various cancers. However, the overexpression of many DNA helicases is required for cancer cell proliferation or resistance to DNA damage [9]. In addition, mutations in genes that promote HDR are frequently observed in several cancers, including the prostate cancer [10]. *Cell-cell junction organization* and *Elastic fibre formation* pathways are also enriched. Elastic fiber are bundles of proteins found in the extracellular matrix. The extracellular matrix is commonly deregulated in cancer and affects the cancer progression, promoting metastasis [11]. Finally, *Fatty acids* pathway has been found to be associated to prostate and in fact several studies demonstrated the association of some fatty acids with prostate cancer risk [12, 13]. Moreover all the pathways enriched only in Core&Peel module include at least one gene with a disease-association in the DisGeNET database and/or DEGs. This highlights how these pathways can have a relevance in the prostate cancer.

Description	p.adjust	Count	Num DisGeNet	DEGs	PMID	Reactome Category
Elastic fibre formation	0.00013	24	4	7	22351925	Extracellular matrix organization
Amplification of signal from the kinetochores	0.0022	36	0	16	28651496	Cell cycle
Resolution of D-loop Structuresthrough Synthesis-Dependent Strand Annealing (SDSA)	0.0032	15	3	1	23842644 20541013	DNA repair
Fatty acids	0.0049	10	0	3	11398173 23843441 16683009	Metabolism
Cell-cell junction organization	0.0087	24	4	4	10688036 23450077	Cell-Cell communication
HDR through Homologous Recombination (HRR)	0.0088	25	3	2	30345412 24027196	DNA repair
G2/M DNA replication checkpoint	0.0123	5	0	2	25621662 18048387	Cell cycle
Interleukin-36 pathway	0.0123	5	0	2	10639195	Immune System
Neurexins and neuroligins	0.0137	20	0	3	29666812 25913192	Neuronal System
ERBB2 Activates PTK6 Signaling	0.0139	8	3	4	31294537	Signal Transduction
AURKA Activation by TPX2	0.0249	27	1	4	22864622 30127868 28147341	Cell cycle
Bile acid and bile salt metabolism	0.0327	18	2	7	23940835	Metabolism
Sodium-coupled sulphate, di- and tri-carboxylate transporters	0.0393	4	0	2	27118869	Transport of small molecules
Nuclear Receptor transcription pathway	0.0413	21	4	3	18301781	gene expression

Table S 10: Summary of the representative pathways of each group detected by ClueGO in prostate cancer. Description: name of the Reactome pathway; p.adjust: adjusted p-value (< 0.05); Count: number of genes enriched in that pathway; Num DisGeNET: number of enriched genes with a prostate-annotation in the DisGeNET database; DEGs: number of enriched genes which are differentially expressed; PMID: literature associated to that pathway; Reactome category: the Reactome category of the corresponding pathway.

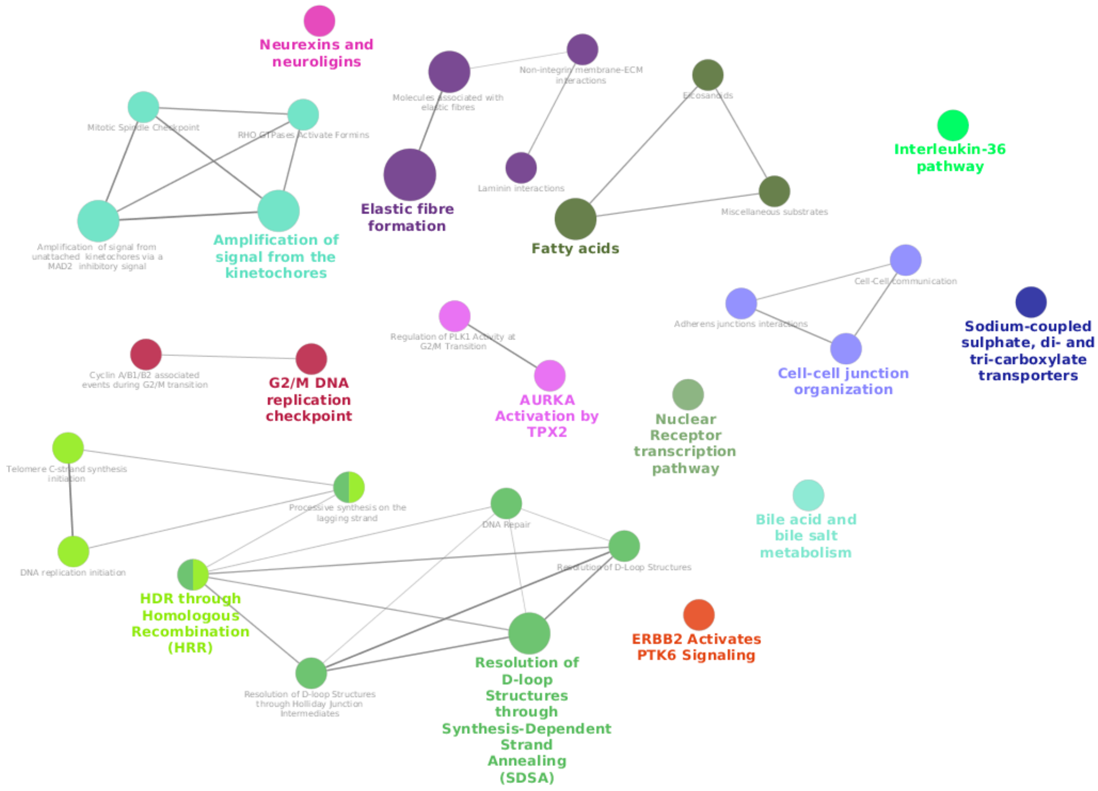

Figure S 13: ClueGO results in prostate cancer case. Each functional group is highlighted by the same color and the pathways belonged to same group are linked with edges. For each group, the description of the representative pathway is colored; the descriptions of the other group members are in grey.

3.3.2 Colorectal cancer

Similarly, all the pathways in colorectal cancer module include genes with disease-association and/or DEGs. Like prostate cancer case, pathways associated to the cell cycle have been found (*G2/M DNA replication checkpoint* and *Telomere C-strand synthesis initiation*). They are mainly involved in DNA replication which represents a crucial point for the genome integrity. A deregulation of this process can lead an accumulation of genetic aberrations that promote diseases such as cancer [14]. Telomeres maintain genome integrity by protecting the end of the chromosome from deterioration. Telomere crisis can cause a wide array of genomic aberrations that can promote cancer progression [15]. Another characteristic of cancer is an altered signal transduction which can lead to uninhibited growth. The *purinergic receptors* pathway is enriched in colorectal module, in fact it has been demonstrated that these receptors, after the binding with the ATP, affect tumor cell growth. In particular, it has been noticed an overexpression of some purienergic receptors in colon cancer [16], along with many malignant tumors. Another enriched pathway involved in the signal transduction is *G alpha signalling events*. Recent findings suggest that the prostaglandin E2, a proinflammatory product, stimulates colon cancer cell growth through a G protein–dependent signaling pathway [17]. Similarly to prostate cancer, fatty acids metabolism is also involved in colorectal cancer. More precisely a rapid metabolism of *arachidonic acid* was reported in various stages of the malignancy, suggesting a possible link between dietary lipids and the incidence of colorectal cancer [18].

**Description**	**p.adjust**	**Count**	**Num DisGeNet**	**DEGs**	**PMID**	**Reactome Category**
Nucleotide-like (purinergic) receptors	0.0004	12	3	5	21484085<br>17391276<br>27321181	Signal Transduction
Crosslinking of collagen fibrils	0.0009	14	1	7	10972180<br>26940881	Extracellular matrix Organization
Arachidonic acid metabolism	0.0033	30	2	12	14619964<br>23715757	Metabolism
Resolution of D-loop Structures through Synthesis-Dependent Strand Annealing (SDSA)	0.0054	17	2	8	23842644	DNA repair
Telomere C-strand synthesis initiation	0.0069	7	1	4	28096526	Cell cycle
Presynaptic depolarization and calcium channel opening	0.0073	10	2	5	17111226	Neuronal System
Defective CHST3 causes SEDCJD	0.0276	7	0	4	16545347	Disease
Intrinsic Pathway of Fibrin Clot Formation	0.0313	14	0	4	23788975<br>8831523	Hemostasis
Regulation of gene expression in beta cells	0.0368	11	0	4		Developmental Biology
Dectin-2 family	0.0368	11	0	5	26379663<br>30231977	Immune System
G2/M DNA replication checkpoint	0.0421	5	0	4	17241873<br>17943134<br>26805514	Cell cycle
G alpha (z) signalling events	0.0438	22	1	12	15837931<br>17145847<br>16432186	Signal Transduction

Table S 11: Summary of the representative pathways of each group detected by ClueGO in colorectal cancer. Description: name of the Reactome pathway; p.adjust: adjusted p-value (< 0.05); Count: number of genes enriched in that pathway; Num DisGeNET: number of enriched genes with a prostate-annotation in the DisGeNET database; DEGs: number of enriched genes which are differentially expressed; PMID: literature associated to that pathway; Reactome category: the Reactome category of the corresponding pathway.

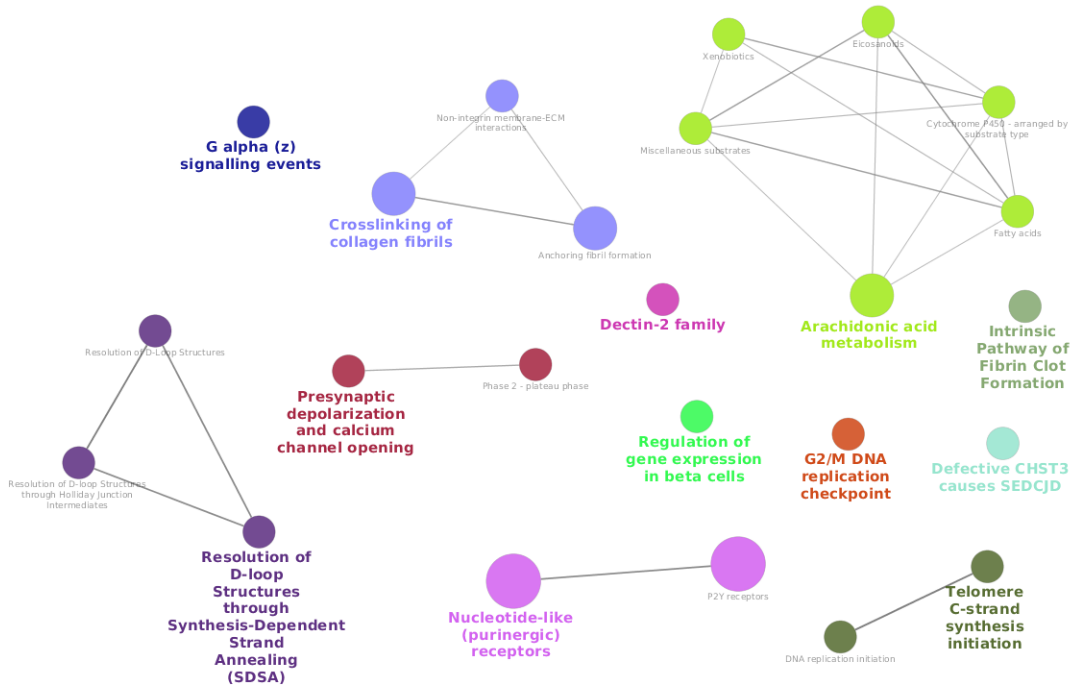

Figure S 14: ClueGO results in colorectal cancer case. Each functional group is highlighted by the same color and the pathways belonged to same group are linked with edges. For each group, the description of the representative pathway is colored; the descriptions of the other group members are in grey.

3.3.3 Rheumatoid arthritis (RA)

Rheumatoid arthritis is a chronic autoimmune disease that primarily affects the lining of the synovial joints. Among all the enriched pathways, two of them (*Neutrophil degranulation* and *Signaling by Interleukins*) are involved in the immune system and include both genes with disease-association (Figure 11b). Neutrophils are the most abundant leukocytes (white blood cells) in mammals and are one of the first-responders of inflammatory cells to migrate towards the site of inflammation. They contribute to RA pathology through the release of cytotoxic and immunoregulatory molecules, promoting the autoimmune processes that underly this disease [19]. The interleukins are proteins expressed by the immune system cells during an immune response. Different studies have demonstrated the involvement of interleukins in RA [20, 21]. In addition, several splice variants of interleukins have been discovered [22], highlighting the involvement of *mRNA splicing* pathway in RA. Other pathways detected only by Core&Peel have a role in immunity and inflammation such as *prefoldin* and *SUMO* proteins. Prefoldin is a family of proteins used in protein folding complexes and seems to function as proinflammatory signals [23]. SUMO is a small ubiquitin-like modifier involved in protein sumoylation, a post-translational-modification event. Sumoylation has been suggested to regulate multiple cellular processes, including inflammation [24].

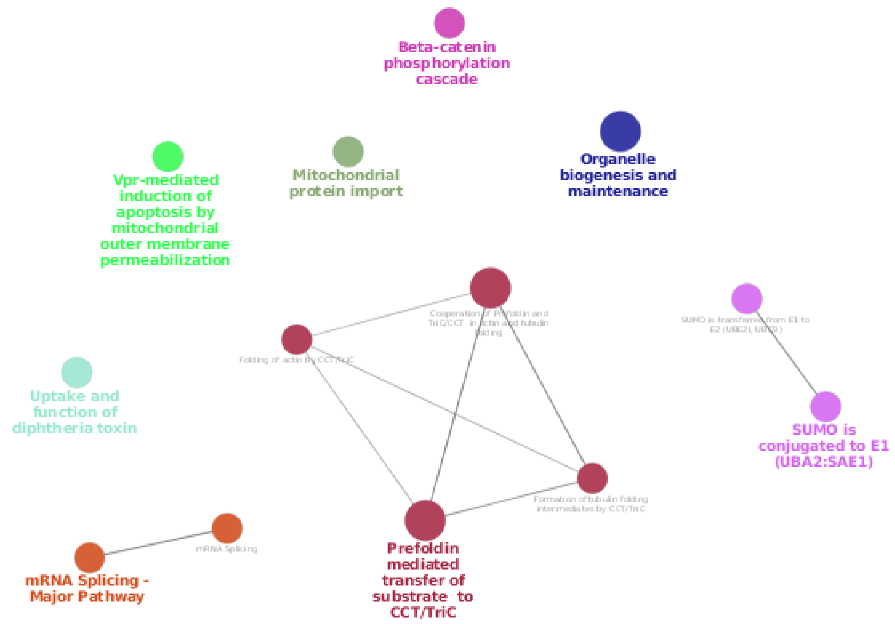

Figure S 15: ClueGO results in rheumatoid arthritis case. Each functional group is highlighted by the same color and the pathways belonged to same group are linked with edges. For each group, the description of the representative pathway is colored; the descriptions of the other group members are in grey.

3.3.4 Asthma

Asthma is a common long-term inflammatory disease of the airways of the lungs. In fact most of the enriched pathways identified only by Core&Peel are involved in the immune system. For example, the interaction between the *Toll-like receptors* (TLRs) and environmental allergens leads to release of various pro-inflammatory mediators from innate cells supporting asthma development [25]. Like in RA, different interleukins have a role in asthma pathogenesis [26, 27]. In addition, *butyrophilins* [28], *leukotrienes and eoxins* are molecules formed in response to inflammatory stimuli. In particular leukotrienes have a role in the pathophysiology of asthma including increased airway smooth muscle activity, microvascular permeability, and airway mucus secretion [29]. Another pathway enriched in asthma module and involved in the immune system is called *TNFs bind their physiological receptors*. TNFs function as cytokine and the binding with their specific receptors has crucial roles in both innate and adaptive immunity. In particular, TNF-α is a proinflammatory cytokine that has been implicated in many aspects of the airway pathology in asthma. Besides, preliminary studies have demonstrated an improvement in asthma quality of life and lung function in patients treated with anti–TNF-α therapy [30]. To conclude, a pathway identified only by Core&Peel which has four genes with disease-association is *G alpha signalling events*. G protein-coupled receptors (GPCRs) regulate numerous airway cell functions, and signaling events transduced by GPCRs are important in both asthma pathogenesis and therapy [31].

Description	p.adjust	Count	num DisGeNet	DEGs	PMID	Reactome category
Prefoldin mediated transfer of substrate to CCT/TriC	0.0029	7	0	1	20445014	Metabolism of proteins
Organelle biogenesis and maintenance	0.0046	25	0	0	27225300 29725325 23466882	Organelle biogenesis And maintenance
Beta-catenin phosphorylation cascade	0.0075	5	0	0	20840015 19683077	Signal Transduction
SUMO is conjugated to E1 (UBA2:SAE1)	0.0097	3	0	0	18755518	Metabolism of proteins
Uptake and function of diphtheria toxin	0.0176	3	0	0		Disease
mRNA Splicing - Major Pathway	0.0247	17	0	0	26160473 21383200	Metabolism of RNA
Mitochondrial protein import	0.0248	8	0	0	15987486	Protein localization
Vpr-mediated induction of apoptosis by mitochondrial outer membrane permeabilization	0.0476	2	0	0	28102843 12813466 12094227	Disease

Table S 12: Summary of the representative pathways of each group detected by ClueGO in rheumatoid arthritis. Description: name of the Reactome pathway; p.adjust: adjusted p-value (< 0.05); Count: number of genes enriched in that pathway; Num DisGeNET: number of enriched genes with a prostate-annotation in the DisGeNET database; DEGs: number of enriched genes which are differentially expressed; PMID: literature associated to that pathway; Reactome category: the Reactome category of the corresponding pathway.

Description	p.adjust	Count	num DisGeNet	DEGs	PMID	Reactome category
TNFs bind their physiological receptors	1.48e-05	14	0	1	18036647 17475560	Immune System
Toll-like Receptor Cascades	0.00011	37	1	2	27543676 28692144	Immune System
G alpha (i) signalling events	0.00016	66	4	5	18278482 18552456	Signal Transduction
Phosphorylation of CD3 and TCR zeta chains	0.00061	10	1	0	99221231 0375551	Immune System
PD-1 signaling	0.00099	10	1	0	18173375 28865147 30644695	Immune System
Other interleukin signaling	0.00597	10	0	0	26750312 15464449 23283176	Immune System
Butyrophilin (BTN) family interactions	0.00686	6	0	0	22030238 20944003	Immune System
Other semaphorin interactions	0.01838	8	0	0	23994348 23932461	Developmental Biology
Synthesis of Leukotrienes (LT) and Eoxins (EX)	0.02707	8	2	0	20920774 21059119	Metabolism

Table S 13: Summary of the representative pathways of each group detected by ClueGO in asthma. Description: name of the Reactome pathway; p.adjust: adjusted p-value (< 0.05); Count: number of genes enriched in that pathway; Num DisGeNET: number of enriched genes with a prostate-annotation in the DisGeNET database; DEGs: number of enriched genes which are differentially expressed; PMID: literature associated to that pathway; Reactome category: the Reactome category of the corresponding pathway.

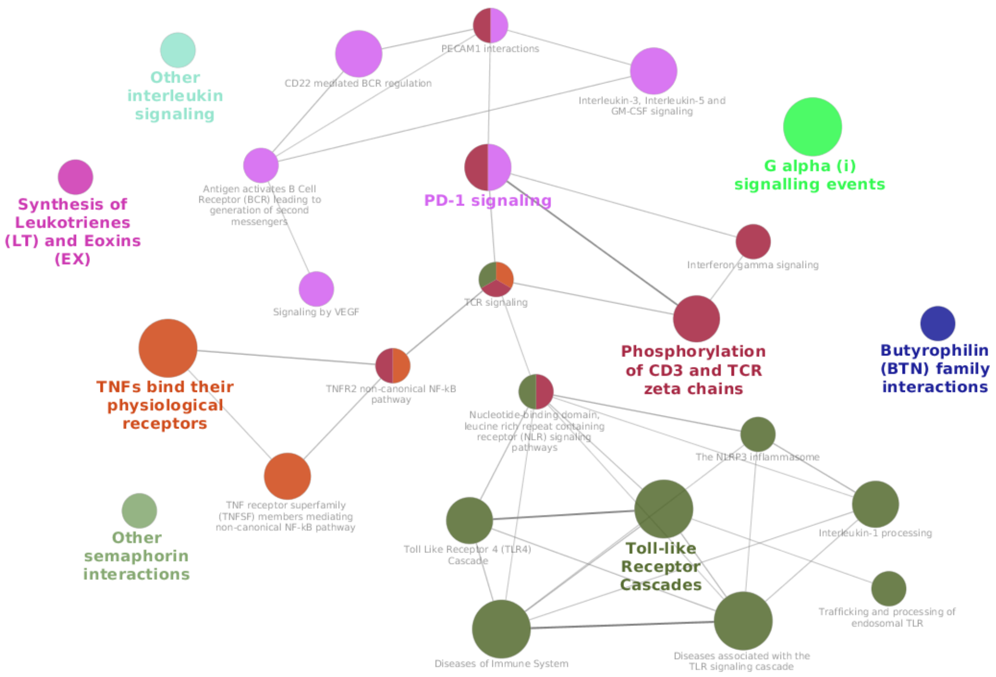

Figure S 16: ClueGO results in asthma case. Each functional group is highlighted by the same color and the pathways belonged to same group are linked with edges. For each group, the description of the representative pathway is colored; the descriptions of the other group members are in grey.

References

[1] H. Nguyen, S. Shrestha, D. Tran, A. Shafi, S. Draghici, and T. Nguyen, "A comprehensive survey of tools and software for active subnetwork identification," *Frontiers in genetics*, vol. 10, p. 155, 2019.

[2] S. Vlaic, T. Conrad, C. Tokarski-Schnelle, M. Gustafsson, U. Dahmen, R. Guthke, and S. Schuster, "Modulediscoverer: identification of regulatory modules in protein-protein interaction networks," *Scientific reports*, vol. 8, no. 1, pp. 1–11, 2018.

[3] N. Alcaraz, H. Kücük, J. Weile, A. Wipat, and J. Baumbach, "Keypathwayminer: detecting case-specific biological pathways using expression data," *Internet Mathematics*, vol. 7, no. 4, pp. 299–313, 2011.

[4] I. Ulitsky, A. Krishnamurthy, R. M. Karp, and R. Shamir, "Degas: de novo discovery of dysregulated pathways in human diseases," *PloS one*, vol. 5, no. 10, 2010.

[5] J. Gu, Y. Chen, S. Li, and Y. Li, "Identification of responsive gene modules by network-based gene clustering and extending: application to inflammation and angiogenesis," *BMC systems biology*, vol. 4, no. 1, p. 47, 2010.

[6] G. Bindea, B. Mlecnik, H. Hackl, P. Charoentong, M. Tosolini, A. Kirilovsky, W.-H. Fridman, F. Pagès, Z. Trajanoski, and J. Galon, "Cluego: a cytoscape plug-in to decipher functionally grouped gene ontology and pathway annotation networks," *Bioinformatics*, vol. 25, no. 8, pp. 1091–1093, 2009.

[7] K. Wang, J. Sun, J. Teng, Y. Yu, D. Zhong, and Y. Fan, "Overexpression of spindle and kinetochore–associated protein 1 contributes to the progression of prostate cancer," *Tumor Biology*, vol. 39, no. 6, p. 1010428317701918, 2017.

[8] J. Zou, R.-Y. Huang, F.-N. Jiang, D.-X. Chen, C. Wang, Z.-D. Han, Y.-X. Liang, and W.-D. Zhong, "Overexpression of tpx2 is associated with progression and prognosis of prostate cancer," *Oncology letters*, vol. 16, no. 3, pp. 2823–2832, 2018.

[9] R. M. Brosh Jr, "Dna helicases involved in dna repair and their roles in cancer," *Nature Reviews Cancer*, vol. 13, no. 8, pp. 542–558, 2013.

[10] C.-C. Chen, W. Feng, P. X. Lim, E. M. Kass, and M. Jasin, "Homology-directed repair and the role of brca1, brca2, and related proteins in genome integrity and cancer," *Annual review of cancer biology*, vol. 2, pp. 313–336, 2018.

[11] P. Lu, V. M. Weaver, and Z. Werb, "The extracellular matrix: a dynamic niche in cancer progression," *Journal of Cell Biology*, vol. 196, no. 4, pp. 395–406, 2012.

[12] L. M. Newcomer, I. B. King, K. G. Wicklund, and J. L. Stanford, "The association of fatty acids with prostate cancer risk," *The Prostate*, vol. 47, no. 4, pp. 262–268, 2001.

[13] T. M. Brasky, A. K. Darke, X. Song, C. M. Tangen, P. J. Goodman, I. M. Thompson, F. L. Meyskens Jr, G. E. Goodman, L. M. Minasian, H. L. Parnes, *et al.*, "Plasma phospholipid fatty acids and prostate cancer risk in the select trial," *Journal of the National Cancer Institute*, vol. 105, no. 15, pp. 1132–1141, 2013.

[14] A.-S. Boyer, D. Walter, and C. S. Sørensen, "Dna replication and cancer: From dysfunctional replication origin activities to therapeutic opportunities," in *Seminars in cancer biology*, vol. 37, pp. 16–25, Elsevier, 2016.

[15] J. Maciejowski and T. de Lange, "Telomeres in cancer: tumour suppression and genome instability," *Nature reviews Molecular cell biology*, vol. 18, no. 3, pp. 175–186, 2017.

[16] G. Nylund, L. Hultman, S. Nordgren, and D. Delbro, "P2y2-and p2y4 purinergic receptors are over-expressed in human colon cancer," *Autonomic and Autacoid Pharmacology*, vol. 27, no. 2, pp. 79–84, 2007.

[17] M. D. Castellone, H. Teramoto, and J. S. Gutkind, "Cyclooxygenase-2 and colorectal cancer chemoprevention: the β-catenin connection," *Cancer research*, vol. 66, no. 23, pp. 11085–11088, 2006.

[18] R. Jones, L.-A. Adel-Alvarez, O. R. Alvarez, R. Broaddus, and S. Das, "Arachidonic acid and colorectal carcinogenesis," *Molecular and cellular biochemistry*, vol. 253, no. 1-2, pp. 141–149, 2003.

[19] H. L. Wright, R. J. Moots, and S. W. Edwards, "The multifactorial role of neutrophils in rheumatoid arthritis," *Nature Reviews Rheumatology*, vol. 10, no. 10, p. 593, 2014.

[20] T. Hirano, T. Matsuda, M. Turner, N. Miyasaka, G. Buchan, B. Tang, K. Sato, M. Shimi, R. Maid, M. Feldmann, *et al.*, "Excessive production of interleukin 6/b cell stimulatory factor-2 in rheumatoid arthritis," *European journal of immunology*, vol. 18, no. 11, pp. 1797–1802, 1988.

[21] M. A. Amin, P. J. Mansfield, A. Pakozdi, P. L. Campbell, S. Ahmed, R. J. Martinez, and A. E. Koch, "Interleukin-18 induces angiogenic factors in rheumatoid arthritis synovial tissue fibroblasts via distinct signaling pathways," *Arthritis & Rheumatism*, vol. 56, no. 6, pp. 1787–1797, 2007.

[22] B. Heinhuis, M. I. Koenders, F. A. van de Loo, M. G. Netea, W. B. van den Berg, and L. A. Joosten, "Inflammation-dependent secretion and splicing of il-32γ in rheumatoid arthritis," *Proceedings of the National Academy of Sciences*, vol. 108, no. 12, pp. 4962–4967, 2011.

[23] B. Henderson and A. G. Pockley, "Molecular chaperones and protein-folding catalysts as intercellular signaling regulators in immunity and inflammation," *Journal of leukocyte biology*, vol. 88, no. 3, pp. 445–462, 2010.

[24] B. Liu and K. Shuai, "Targeting the pias1 sumo ligase pathway to control inflammation," *Trends in pharmacological sciences*, vol. 29, no. 10, pp. 505–509, 2008.

[25] A. Zakeri and F. G. Yazdi, "Toll-like receptor-mediated involvement of innate immune cells in asthma disease," *Biochimica et Biophysica Acta (BBA)-General Subjects*, vol. 1861, no. 1, pp. 3270–3277, 2017.

[26] E. L. Rael and R. F. Lockey, "Interleukin-13 signaling and its role in asthma," *World Allergy Organization Journal*, vol. 4, no. 3, pp. 54–64, 2011.

[27] T. A. Chatila, "Interleukin-4 receptor signaling pathways in asthma pathogenesis," *Trends in molecular medicine*, vol. 10, no. 10, pp. 493–499, 2004.

[28] L. Abeler-Dörner, M. Swamy, G. Williams, A. C. Hayday, and A. Bas, "Butyrophilins: an emerging family of immune regulators," *Trends in immunology*, vol. 33, no. 1, pp. 34–41, 2012.

[29] P. Montuschi and M. Peters-Golden, "Leukotriene modifiers for asthma treatment," *Clinical & Experimental Allergy*, vol. 40, no. 12, pp. 1732–1741, 2010.

[30] C. Brightling, M. Berry, and Y. Amrani, "Targeting tnf-α: a novel therapeutic approach for asthma," *Journal of Allergy and Clinical Immunology*, vol. 121, no. 1, pp. 5–10, 2008.

[31] R. B. Penn, "Embracing emerging paradigms of g protein-coupled receptor agonism and signaling to address airway smooth muscle pathobiology in asthma," *Naunyn-Schmiedeberg's archives of pharmacology*, vol. 378, no. 2, pp. 149–169, 2008.
